# Glucose Modulates Marine Xylanase Activity: Insights From *Caulerpa lentillifera* and Synthetic β‐1,3‐glucoxylans

**DOI:** 10.1002/cbic.70406

**Published:** 2026-07-10

**Authors:** Nils H. Rustmeier, Nitish Verma, Fabian Pfrengle

**Affiliations:** ^1^ Institute of Organic Chemistry, Department of Natural Sciences and Sustainable Resources BOKU University Vienna Austria

**Keywords:** automated glycan assembly, *Caulerpa lentillifera*, glycobiology, marine xylanase, xylan

## Abstract

Marine xylans are major cell‐wall constituents of green and red algae. While the β‐1,4 and β‐1,3/β‐1,4 mixed‐linkage xylans (MLX) of red algae are homopolymers of xylose, several studies have reported glucose incorporation into green algal β‐1,3‐xylans. However, the consequences of intrachain glucose insertions for the degradation of β‐1,3‐xylan by endo‐acting xylanases remain unknown. Here, high‐performance liquid chromatography coupled with mass spectrometry (HPLC‐MS) analyses demonstrate that glucose is an integral part of di‐ and trisaccharides released from the xylan of green alga *Caulerpa lentillifera* upon treatment with a β‐1,3‐xylanase and a mixed‐linkage xylanase (MLXase) from marine bacteria. Cleavage patterns on synthetic glucoxylan oligosaccharides generated by automated glycan assembly show that the β‐1,3‐xylanase hydrolyzes the β‐1,3‐bond between glucose and xylose, revealing a previously unrecognized activity within the glycoside hydrolase family 26.

## Graphical abstract

Two recombinant xylanases from marine bacteria, β‐1,3‐xylanase Xyl4 and mixed‐linkage xylanase AlXyn26A, were used to digest glucoxylan structures extracted from the green alga *Caulerpa lentillifera* and synthesized by automated glycan assembly. High‐performance liquid chromatography coupled with mass spectrometry (HPLC‐MS) analysis of the reaction products determined glucose‐modulated hydrolysis patterns and subsite specificities, revealing a previously unrecognized cleavage site.

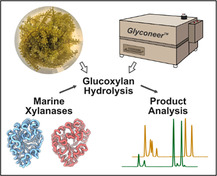

## 1 Introduction

Marine algae fix gigatons of CO_2_ into biomass each year while not competing for scarce agricultural land. Still, algal biomass is utilized at a relatively small scale, primarily for human consumption as foods (e.g., nori) and food ingredients such as hydrocolloids (carrageenan, alginate, agar) [1, 2]. Algal ingestion has been linked to health benefits due to anticoagulant, anti‐inflammatory, and antitumor effects [3, 4, 5, 6, 7, 8]. Given the high carbohydrate content of ~50% of dry weight [9], algae also represent a promising sustainable resource. Yet, the conversion of algal biomass into industrial feedstocks remains underexplored, partially due to limited availability of well‐characterized carbohydrolytic enzymes [10, 11].

The siphonous green algae of the genus *Caulerpa* contain β‐1,3‐xylan as major cell wall polysaccharide [12]. Most sources describe *Caulerpa* xylans as linear, purely β‐1,3‐linked xylose polymers. However, numerous studies also document alternative linkages and minor amounts of other monosaccharide components. In particular, reports on the glucose content in β‐1,3‐xylan range from essentially undetectable in *C. brachypus* and *C. filiformis* [12, 13], to around 5% in *C. racemosa* and *C. lentillifera* [7, 14, 15], and up to 10% in *C. anceps* [16]. Monoxylosyl branches have been described at approximately 12% and 8% of backbone *O*−4 positions in *C. racemosa* and *C. lentillifera*, respectively [15, 17]. Taken together, *Caulerpa* β‐1,3‐xylan is heterogeneous, with variations likely influenced by season and habitat [18].

Algae are hosts to a diverse panel of marine bacteria, acting as their primary carbon source. Structural diversity of carbohydrates as seen in the β‐1,3‐xylan further motivates analyses of enzymatic specificities that enable salvaging by microorganisms. The degradation of green algal β‐1,3‐xylan by β‐1,3‐xylan hydrolases (β‐1,3‐xylanases) is relatively well studied. The β‐1,3‐xylanase Xyl4—originating from the marine bacterium *Vibrio* sp. AX‐4—was among the first biochemically characterized enzymes acting on green algae xylans [19, 20]. Its recombinant catalytic domain has since become a standard tool to produce xylooligosaccharides (XOS) from β‐1,3‐xylan in research [21, 22]. Xyl4 is an endo‐acting xylanase from glycoside hydrolase family 26 (GH26), which cleaves internal linkages within the β‐1,3‐xylose polymer. Structural investigations of Xyl4’s active site have highlighted substrate‐binding subsites spanning at least −3 to +2 [23]. Favorable occupancy of the −3 subsite aligns with xylotriose being the predominant product of Xyl4 action on β‐1,3‐xylan [19]. By contrast, the mixed‐linkage xylan (MLX) of red algae appears to be a poor substrate for all investigated β‐1,3‐xylanases, based on the limited data currently available [24]. MLX degradation is mediated by specialized MLX‐hydrolyzing enzymes from GH26 termed MLXases. These more recently discovered enzymes are virtually incapable of digesting natural β‐1,3‐xylan, as shown for the polysaccharide extracted from the green alga *C. lentillifera* [25]. However, we previously demonstrated that the recombinant MLXases AlXyn26A and MfXyn26A can degrade purely β‐1,3‐linked XOS produced by automated glycan assembly (AGA) [26].

Collectively, marine xylan depolymerization into XOS is well studied, but the impact of nonxylose residues in β‐1,3‐xylan remains poorly defined. Prior work suggests that glucose‐enriched *C. racemosa* hydrolysates impede Xyl4‐mediated degradation [14]. Here, we investigate the role of glucose within β‐1,3‐xylan chains during enzymatic digestion using natural β‐1,3‐xylan from *C. lentillifera* as well as synthetic glucoxylan oligosaccharides (GXOS) bearing glucose substitutions at defined positions along the β‐1,3‐linked backbone. Three hexasaccharides were synthesized by AGA and subjected to degradation by Xyl4 and AlXyn26A to dissect subsite constraints and product pathways. By mapping enzymatic digestion and substrate preferences across this defined GXOS panel, we establish substrate rules that inform enzyme selection for algal xylan bioprocessing.

## 2 Materials and Methods

### 2.1 Materials

Dehydrated edible *C. lentillifera* (grown in Vietnam) were ordered from an online marketplace. Solvents and additives for high‐performance liquid chromatography coupled with mass spectrometry (HPLC‐MS) were LC–MS grade unless otherwise noted. Acetonitrile (ACN), ammonia (25% solution, analytical grade), and ethanol were from Fisher Chemical and sodium hydroxide from Thermo Scientific. Sodium borohydride, formic acid, chloroform, and acetone were from Sigma–Aldrich and methanol from Honeywell. Glacial acetic acid (AcOH) was from Roth. Ultrapure water was used for all purifications, dialyses, and liquid chromatography.

### 2.2 Xylan Extraction

The extraction of β‐1,3‐xylan was based on an established protocol [15]. Dehydrated *C. lentillifera* material was first soaked in excess deionized water for 1 h at room temperature. A portion (~14 g wet weight) was then frozen in liquid nitrogen and ground on dry ice with a prechilled mortar and pestle. The ground material was lyophilized for 72 h and stored at room temperature until extraction.

Ground, dried algae (~0.30 g) were sequentially defatted at room temperature under stirring with 50 mL of 80% (v/v) ethanol, 1:1 (v/v) methanol/chloroform, and acetone (each step 30 min; one extraction per solvent). Solids were collected in a 150 mm cellulose filter (5–8 µM pore size, Labsolute), air‐dried, and then rehydrated in 100 mL water. The water‐soluble fraction was extracted at 80°C for 4 h in a thermal mixer (800 rpm), and the suspension was vacuum‐filtered through a 47 mm nylon filter membrane (0.45 µm pore size, Millipore). The retained residue was washed with excess water to remove soluble components.

Xylan was extracted from the washed residue with 30 mL of 24% (w/v) KOH containing 0.1% (w/v) NaBH_4_ for 2 h at room temperature while rotating (10 rpm). The extract was clarified by centrifugation (14,000 rcf, 15 min, 4°C) and neutralized to pH ~ 7 with glacial acetic acid. For xylan precipitation, 200 mL of cold absolute ethanol was added to the neutralized extract, mixed, and incubated at 4°C overnight. The fibrous, faintly green precipitate was collected via centrifugation (14,000 rcf, 15 min, 4°C) and lyophilized.

The resulting crude β‐1,3‐xylan was desalted by dialysis (MWCO 3.5 kDa, 22 mm SnakeSkin tubing, Thermo Fisher) against a 20,000‐fold excess (w/w) of water at 4°C with multiple changes over 72 h and finally dried by vacuum centrifugation at room temperature.

### 2.3 Production and Characterization of Xylanases

Recombinantly expressed xylanases Xyl4 and AlXyn26A were prepared as described previously and stored at −20°C until deployment [26]. Specific activities were determined by a reducing‐sugar assay adapted from Zhao et al. (2023) with minor modifications [25]. Briefly, 10 µL of undiluted enzyme was mixed with 90 µL of 0.5% (w/v) xylan (β‐1,3‐xylan for Xyl4 and MLX for AlXyn26A) and incubated at 37°C for 10 min. The reaction was stopped by addition of 100 µL DNS reagent (10 g/L 3,5‐dinitrosalicylic acid, 30 g/L sodium potassium tartrate, and 16 g/L sodium hydroxide in water), followed by color development at 100°C for 10 min. The absorbance at 540 nm was measured with a cuvette spectrophotometer (*d* = 1 cm) and calibrated using a d‐xylose standard prepared under identical conditions without enzyme (Figure S3). Enzymatic activity was defined as xylose equivalents (μmol) released per minute (U), and specific activity as U per mg of enzyme (Table S1).

### 2.4 Xylanase Assays

Xylanase reactions were performed as described previously [26]. Substrates (extracted β‐1,3‐xylan or synthetic GXOS) were prepared at 0.1% (w/v) in assay buffer (20 mM Tris‐HCl, pH 7.4, 200 mM NaCl) to a total reaction volume of 150 µL. Xyl4 and AlXyn26A were used at 1 µM, with batch‐specific activities of 1.9 ± 0.9 U/mg and 2.4 ± 0.2 U/mg against β‐1,3‐xylan and MLX, respectively. Reactions were incubated in a thermal mixer (Eppendorf) at 650 rpm under temperature control, with early time points taken after 1 h at 20°C and endpoint samples after 24 h at 37°C. Reactions were terminated by heating at 85°C for 15 min and stored at −20°C until purification. For natural xylan extracts, no‐enzyme controls were processed in parallel. Enzyme reactions and analyses with synthetic GXOS as substrates were performed in technical duplicate (*n* = 2).

### 2.5 Product Purification

Reaction samples (75 µL) were thawed and purified using Thermo Scientific HyperSep Hypercarb SPE 96‐well cartridges (25 mg bed per well). Using 1‐min centrifugation steps at 100 rcf, cartridges were conditioned with 1 × 450 µL of 100% methanol and equilibrated with 3 × 450 µL of 80 mM ammonium formate buffer (pH 3, “FA buffer”). Samples were loaded (2 min at 100 rcf) and washed with 3 × 450 µL FA buffer. Bound oligosaccharides were eluted stepwise with 1 × 450 µL of 65% ACN (v/v) in FA buffer and 1 × 450 µL of 100% ACN. Eluates were combined and dried in a vacuum centrifuge at ≤30°C.

### 2.6 High‐Performance Liquid Chromatography coupled with Mass Spectrometry (HPLC‐MS)

For product analysis, dried samples were reconstituted in 20 µL water and analyzed by porous graphitic carbon (PGC) chromatography on a Thermo Scientific Hypercarb column (100  ×  4.6 mm, 5 µm particle size). The HPLC system was a Shimadzu LC‐10 coupled to dual detection with a Shimadzu LCMS‐2020 mass spectrometer (electrospray ionization, positive and negative modes) and an Alltech 3300 evaporative light‐scattering detector (ELSD, drift tube temperature = 60°C, receiver gain = 2).

After equilibrating the column at 2.5% (v/v) acetonitrile (ACN) in water + 0.1% AcOH, 17 µL of sample was injected. Separations were performed at room temperature and 0.7 mL/min under the following program: isocratic at 2.5% (v/v) ACN for 8 min; linear gradient to 40% (v/v) ACN over 25 min; ramp to 100% ACN over 4 min; hold at 100% ACN for 4 min; re‐equilibrate to 2.5% (v/v) ACN over 2 min.

### 2.7 General Methods for Synthesis

All purchased chemicals were used without further purification. Solvents were dried over activated 4 Å molecular sieves. Aqueous solutions of salts were saturated unless stated otherwise. The concentration of organic solutions was performed under reduced pressure at 40°C, unless stated otherwise. All reactions were monitored by thin layer chromatography (unless stated otherwise), which was performed on precoated plates (5 × 10 cm, 0.25 mm layer thickness, Silica Gel 60F_254_, Merck). Spots were detected by a UV‐lamp (254 nm) and then by dipping reagent (anisaldehyde‐H_2_SO_4_ or Hanessian’s stain) and heating at 250°C using a hotplate. Filtrations were performed using 25 mm syringe filters (PTFE, 0.45 μm, Fisher Brand). Silica gel (0.040–0.063 mm, Macherey‐Nagel) was used for normal phase column chromatography, and purifications were performed either by hand or on the automatic system Interchim puriFlash 4125 or Interchim puriFlash 5.250. HPLC‐MS was performed on the system described above. NMR spectra were recorded on a Brucker 600’54 Ascend Evo with Prodigy CPP 1.1 BBO 600 S3 (600 MHz for ^1^H, 151 MHz for decoupled ^13^C) using standard software provided by the manufacturer. ^1^H spectra were referenced to 0 (external calibration to TMS) or 2.05 ppm for solutions in (CD_3_)_2_CO, 0 ppm (external calibration to DSS) for solutions in D_2_O; ^13^C spectra were referenced to 29.84 ppm for solutions in (CD_3_)_2_CO and 67.19 ppm (external calibration to 1,4‐dioxane) for solutions in D_2_O. Assignments are based on 2D ^1^H‐^1^H COSY, ^1^H‐^13^C HSQC, ^1^H‐^13^C CLIP‐HSQC, ^1^H‐^13^C HMBC/H2BC, and ^1^H‐^1^H TOCSY spectra. Peaks of the respective glycosyl residues in the ^1^H NMR spectrum are labeled alphabetically (A, B, C, D, etc.) starting from the reducing end. Electrospray ionization ‐ high‐resolution mass spectrometry data were obtained using samples dissolved in ACN/H_2_O on an Agilent Technologies 6230B LCMS‐TOF or a Waters Xevo G2‐XS QTof instrument. Datasets were analyzed by Mass Hunter Qualitative Navigator B.08.00 software or mass‐adducts were calculated with Mass Hunter Isotope Distribution Calculator v. 8.0.8208.0 software. AGA was performed on a Glyconeer 3.1 with software provided by the manufacturer. Linker‐functionalized resin was purchased from GlycoUniverse.

### 2.8 Synthesizer Modules and Conditions

Linker‐functionalized resin (12.5 μmol of hydroxyl groups) was placed in the reaction vessel of the Glyconeer 3.1 and swollen for at least 30 min in DCM. Before every reaction step, the resin was washed with DMF and DCM. Subsequently, the glycosylation (Module A), capping (Module B), and Fmoc deprotection (Module C) steps were performed. Mixing of the components was accomplished by bubbling Argon through the reaction mixture. The settings for the different modules in the manufacturer’s program were used as provided by GlycoUniverse.

### 2.9 Module A: Glycosylation with Glycosyl Phosphates

Resin (12.5 μmol of hydroxyl groups) was swollen in DCM and the temperature of the reaction vessel was adjusted according to each AGA reaction (please see experimental procedure in the SI). Prior to the glycosylation reaction, the resin was washed with 62 mM TMSOTf in DCM and then DCM only. For the glycosylation reaction, the DCM was drained, and a solution of phosphate BB (5 equiv., 60 mM DCM) was delivered to the reaction vessel at low temperature (see individual reaction in SI). The reaction was initiated by the addition of 62 mM TMSOTf in DCM (1 mL). The glycosylation was performed for 5 min at −35°C and then for 30 min at −20°C. Subsequently, the solution was drained, and the resin was washed 3 times with DCM at 25°C.

### 2.10 Module B: Capping

The temperature of the reaction vessel was adjusted to 30°C. 10% (v/v) pyridine in dry DMF (2 mL) was delivered. After 3 min, the reaction solution was drained, and the resin was washed with DCM (3 times with 3 mL). Then a solution of 10% (v/v) acetic anhydride and 2% (v/v) methanesulfonic acid in DCM (2 mL) was delivered to the reaction vessel. After 10 min, the solution was drained, and the resin was washed with DCM (3 times with 3 mL). Two capping cycles were performed per glycosylation.

### 2.11 Module C: Fmoc Deprotection

The resin was washed with DMF, swollen in DMF, and the temperature of the reaction vessel was adjusted to 20°C. Prior to the deprotection reaction, DMF was drained, and the resin was washed with DMF 3 times. For Fmoc deprotection, 2 mL of a solution of 20% (v/v) piperidine in DMF was delivered to the reaction vessel. After 10 min, the reaction solution was drained, and the resin was washed with DMF (3 times with 3 mL) and DCM (3 times each with 3 mL). The temperature of the reaction vessel was decreased to −20°C for the next step.

### 2.12 Cleavage from Solid Support

After assembly of the oligosaccharides, cleavage from solid support was accomplished by UV irradiation at 305 nm in a continuous flow photoreactor (photocleavage device: Easy‐PhotoChem V3 UV‐150 from Vapourtec E‐series with attached syringe pump) as previously described [27].

### 2.13 Global Deprotection

To a solution of protected hexasaccharide (1 equiv.) in THF, a solution of 0.5 M NaOMe in MeOH (15 equiv.) was added at RT, and the reaction mixture was stirred overnight. After confirmation of reaction completion by TLC, the reaction mixture was neutralized by the addition of IR‐120 H^+^ resin while stirring. Then, the reaction mixture was filtered, and the filtrate was concentrated under reduced pressure to yield a partially deprotected crude, which was kept under high vacuum until usage in the next step without any further purification. To a solution of partially deprotected crude in *t*‐BuOH, H_2_O, and AcOH, unreduced 10% Pd/C (1.4–1.5 × w/w of the protected hexasaccharide) was added, and the reaction mixture was stirred in the H_2_ reactor under a pressure of 8 bar H_2_. After reaction completion as observed by HPLC‐MS or matrix‐assisted laser desorption/ionization mass spectrometry, the reaction mixture was filtered using a PTFE syringe filter (0.45 µm) and concentrated under reduced pressure to yield a crude product, which was purified using prepacked C18 (500 mg, 6 mL, Waters Sep‐Pak) column chromatography with 0%−20% (v/v) ACN in water +0.1% (v/v) AcOH to obtain the pure product.

## 3 Results and Discussion

### 3.1 Activity of Recombinant Xylanases on β‐1,3‐xylan Extract

**FIGURE 1:**
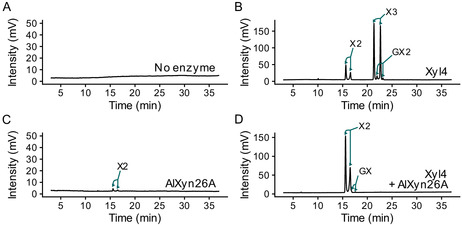
Endpoint product HPLC‐MS analyses after enzymatic incubation of *C. lentillifera* β‐1,3‐xylan. Shown are the ELSD intensities (Millivolt, mV) versus retention time (min). Peak assignment was supported by ESI‐MS (see Figure S1). (A) The mock‐treated control without added enzymes shows no oligosaccharide release. (B) Incubation of β‐1,3‐xylan with 1 µM Xyl4 gives rise to all‐β‐1,3‐linked xylo‐trisaccharide (X3), gluco‐dixylo‐trisaccharide (GX2), and xylo‐disaccharide (X2). (C) 1 µM AlXyn26A incubation produces minor amounts of X2. (D) Coincubation with 1 µM Xyl4 and 1 µM AlXyn26A predominantly produces X2 with trace amounts of gluco‐xylo‐disaccharide (GX).

To probe backbone composition and potential glucose incorporation, we analyzed β‐1,3‐xylan extract from *C. lentillifera* using recombinant xylanases and a previously reported product‐detection pipeline [26]. The β‐1,3‐xylan extract (~1% yield) was first confirmed to be exclusively polymeric, as no low‐molecular‐weight contaminants were detected in mock‐treated controls (Figure 1A). We then assessed the release of glucose‐bearing oligosaccharides upon treatment with recombinant β‐1,3‐xylanase Xyl4. ELSD revealed three products with retention times (*t*_R_) between 15 and 25 min, which were assigned to β‐1,3‐xylo‐disaccharide (X2, *t*_R_ ≈ 16 min), β‐1,3‐xylo‐trisaccharide (X3, major peak at *t*_R_ ≈ 22 min), and a trisaccharide comprising one hexose (interpreted as glucose) and two xyloses (GX2, *t*_R_ ≈ 23 min) by ESI‐MS (Figure 1B). All corresponding reaction‐endpoint ion chromatograms are provided in Figure S1. The relative ELSD intensities indicate that xylose‐only products vastly predominate, consistent with reports of up to ~5% total glucose in *C. lentillifera* β‐1,3‐xylan [7].

The observed products align with the results obtained by Kiyohara et al. (2006), who used Xyl4 to generate XOS from *C. racemosa* and identified an all‐β‐1,3 Glc–Xyl–Xyl trisaccharide by MALDI‐TOF MS [14]. Our GX2 signal matches this composition, indicating that the same monosaccharide sequence was obtained. Mechanistically, the release of Glc–Xyl–Xyl implies glucose tolerance at Xyl4 subsites −3 and +1, while the enzyme retains specificity for β‐1,3 linkages.

We next assessed the activity of the mixed‐linkage xylanase (MLXase) AlXyn26A on β‐1,3‐xylan. Consistent with prior work reporting a digestion rate of ~0.3% on this substrate relative to mixed‐linkage xylan (MLX) [7], only trace amounts of X2 were released during our experiments (Figure 1C). However, in combined‐enzyme reactions on β‐1,3‐xylan using both Xyl4 and MLXase, the Xyl4‐generated trisaccharides (X3 and GX2) were not detected by ELSD. Instead, X2 became the major product, with minor amounts gluco‐xylo‐disaccharide (GX, *t*_R_ ≈ 17 min) traceable (Figure 1D). This product pattern is consistent with AlXyn26A‐catalyzed secondary cleavage of X3 and GX2 and indicates that its active site requires a β‐1,3‐linked xylose located at subsite −1 while tolerating glucose at subsite −2.

While these observations confirm glucose content within *Caulerpa lentillifera* xylan and identify glucose‐tolerant positions during polymer hydrolysis, they do not explain AlXyn26A’s reluctance to degrade extracted β‐1,3‐xylan. Structural analysis shows that AlXyn26A (PDB: 7XJR, 7XS3) has a tunnel‐shaped active site requiring substrate threading [25]. Natural β‐1,3‐xylan, however, forms microfibrils of intertwined triple helices stabilized by an extensive hydrogen‐bond network [28, 29, 30]. Given this architecture, accessible single β‐1,3‐xylan strands capable of threading are likely scarce, accounting for the minor X2 yields observed after extended incubation. By contrast, Xyl4 features an open‐groove active site (PDB: 2DDX, 3VPL) that can engage the microfibril at internal positions [20, 23].

### 3.2 AGA of Glucoxylan Hexasaccharides

**SCHEME 1:**
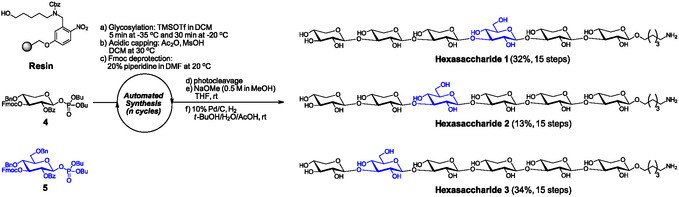
AGA and deprotection reactions for the synthesis of glucoxylan hexasaccharides.

To further investigate the patterns of glucoxylan engagement by the xylanases, three all‐β‐1,3‐linked glucoxylan hexasaccharides (**1**–**3**), with glucose residues in distinct positions, were prepared using AGA as a powerful tool to rapidly synthesize large numbers of oligosaccharide products from a limited set of monosaccharide building blocks (BBs) [31]. For synthesis of the glucoxylan hexasaccharides, xylose phosphate donor **4** [26], suitable for the synthesis of β‐1,3‐xylans, and glucose phosphate donor **5** [32], suitable for synthesis of β‐1,3‐glucans, were used (Scheme 1). A fluorenylmethyloxycarbonyl (Fmoc) group served as an orthogonal temporary protecting group for oligosaccharide chain elongation at the C3‐position, while a benzoyl (Bz) group at the C2‐position ensured β‐stereoselectivity in the glycosidic bond formation through neighboring group participation. The remaining positions were permanently protected with benzyl (Bn) groups. The AGA reactions were performed on a Merrifield resin, modified with a photocleavable linker. To maximize the glycosylation yield for the glucosylations, two coupling cycles were performed, while one coupling cycle was sufficient for the xylosylations.

Following assembly, photocleavage, and HPLC‐purification, the protected hexasaccharides were globally deprotected by Zemplén transesterification using NaOMe to remove the ester‐type Bz groups and catalytic hydrogenolysis to remove the Bn and Cbz groups. The final hexasaccharides (**1**–**3**), equipped with an aminopentyl linker, were obtained after C18 column chromatography (see Supporting Information for details).

### 3.3 Activity of Recombinant Xylanases on Synthetic Glucoxylan Hexasaccharides

**FIGURE 2:**
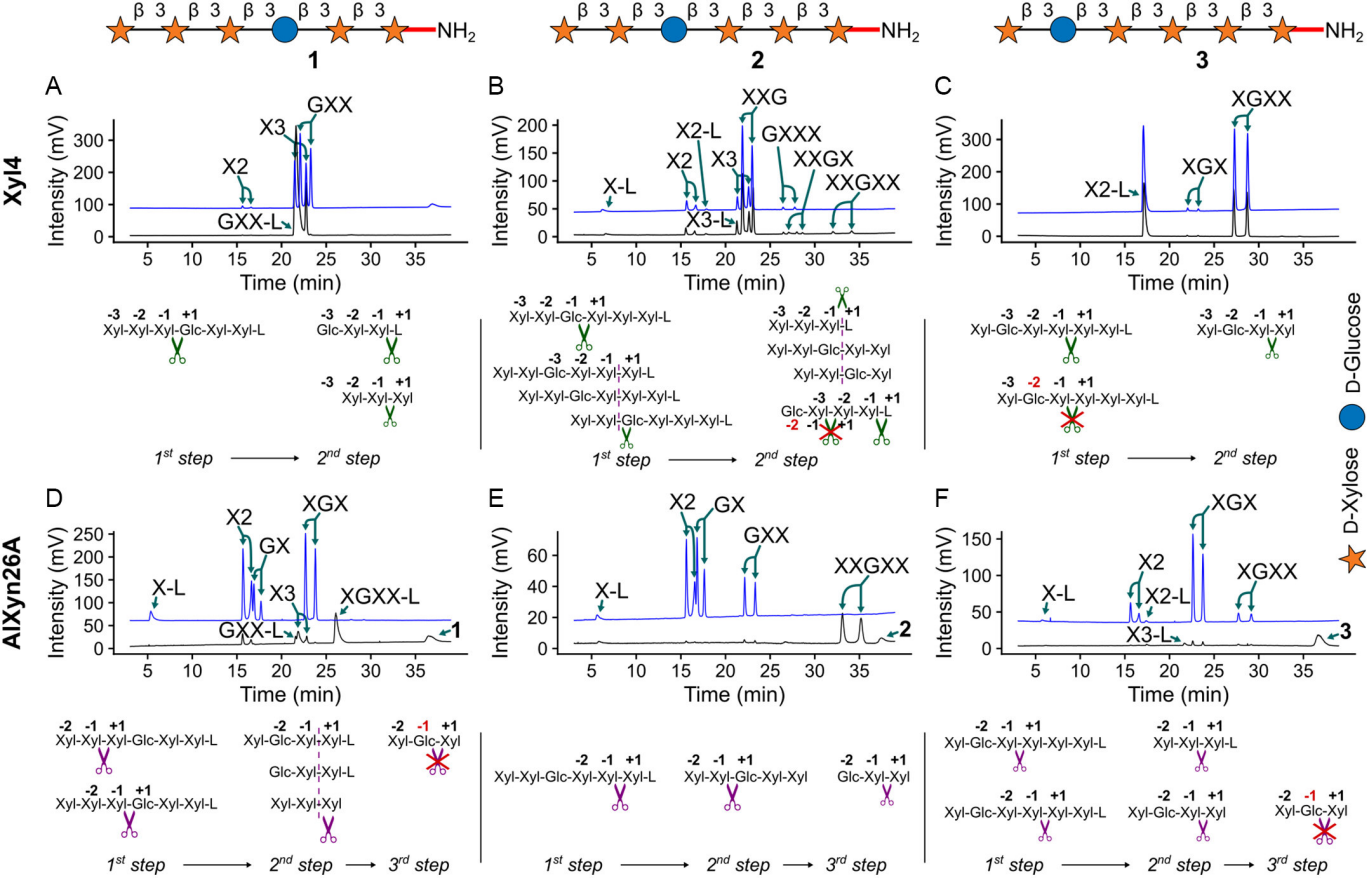
HPLC product profiles of synthetic β‐1,3‐GXOS digests and inferred stepwise degradation pathways. Symbolic representations of hexasaccharides **1**–**3** are shown at the top. Chromatograms in A–F in black are for products after 1 h at 20°C and in blue for products after 24 h at 37°C, plotted as ELSD signal (mV) versus retention time (min). Peak assignments were supported by ESI‐MS (see Figure S1). All‐β‐1,3‐linked oligosaccharide sequences are in one‐letter code (from nonreducing to reducing ends) with “X” for d‐xylose and “G” for d‐glucose. Xylo‐disaccharide and ‐trisaccharide are designated “X2” and “X3,” respectively. Linker‐functionalization is indicated by “‐L.” Below the chromatograms, the proposed sequential cleavage events are shown. Scissor icons mark hydrolysis sites and are scaled to reflect relative reaction rates. Numerals above each oligosaccharide sequences denote enzyme subsite occupancy. Crossed‐out scissors and red subsite numbers indicate disfavored reactions.

After confirming the release of glucose‐containing oligosaccharide from natural *C. lentillifera* β‐1,3‐xylan, we sought to define the position‐dependent effects of glucose incorporation on Xyl4‐ and AlXyn26A‐catalyzed hydrolysis of the three synthetic glucoxylan hexasaccharides **1**–**3**. These reactions, including substrate structures, product profiles, enzymatic subsite assignments, and annotated cleavage sites are summarized in Figure 2.

Analysis of Xyl4 acting on hexasaccharide **1** shows that glucose is well tolerated at subsite +1, with the two trisaccharides XXX and GXX‐L (X = d‐xylose, G = d‐glucose, ‐L = aminopentyl linker) released as the primary products. During subsequent engagement of GXX‐L, with glucose at subsite −3, Xyl4 also cleaved the glycosidic bond to the linker in a secondary reaction, yielding GXX. From XXX, trace amounts of X2 were released (Figure 2A). The reaction of Xyl4 with hexasaccharide **2** also yielded two predominant trisaccharides, XXX‐L and XXG, demonstrating hydrolysis of the β‐1,3 Glc–Xyl linkage with glucose at subsite −1. Additional reactions producing XXGXX and the tetrasaccharides GXXX‐L and XXGX progressed more slowly, resulting in minor signals after 1 h. After 24 h, only linker‐free GXXX (with glucose at subsite −2) was detected, whereas XXGXX and XXGX were depleted by further enzymatic degradation (Figure 2B). The activity of Xyl4 on hexasaccharide **3** further demonstrates that glucose is well tolerated at subsite −3, as evidenced by the release of XGXX and the linker‐bearing X2‐L. By contrast, hydrolysis yielding trisaccharide XGX remains highly disfavored with glucose at subsite −2, likely due to steric clashes introduced by the additional hydroxymethyl group of glucose relative to xylose (Figure 2C).

Collectively, these results show that glucose positioning across subsites (−3 to +1) governs hydrolysis site selection. The reaction patterns, including cleavage of the β‐1,3 Xyl–Glc linkage in hexasaccharide **1**, align with the reported Xyl4‐catalyzed formation of the all‐β‐1,3‐linked GXX trisaccharide from natural β‐1,3‐xylan [14]. By contrast, cleavage of the β‐1,3 Glc–Xyl linkage observed in hexasaccharide **2** represents a novel activity. This reaction yielded two trisaccharides, which are, owing to favorable subsite occupancy, the main products of Xyl4 activity. Whether a similar reaction occurs in nature, and whether other GH26 β‐1,3‐xylanases share or even exceed this capability, remains to be determined. Notably, in the marine bacterium *Vibrio* sp. EA‐2, two active β‐1,3‐xylanases were recently described [33]. Encoded within the same xylan utilization locus, EA‐2‐Xyn26B (>90% sequence identity with Xyl4) is essential for bacterial growth on *C. lentillifera* β‐1,3‐xylan as the sole organic carbon source, whereas EA‐2‐Xyn26A is dispensable, suggesting a complementary role potentially targeting glucose substitutions or other structural features of the β‐1,3‐xylan.

Previously, we showed that the MLXase AlXyn26A, usually targeting β‐1,4 glycosidic bonds, can degrade purely β‐1,3‐linked synthetic XOS into disaccharides after extended incubation. Here, the product profiles from hexasaccharides **1**–**3** likewise comprise mainly disaccharides and indicate that β‐1,3‐linked glucose is tolerated at subsites −2 and +1, but not at −1. Consequently, the trisaccharide XGX accumulated when **1** or **3** served as substrates (Figure 2D–F). Moreover, the AlXyn26A‐catalyzed reactions proceeded more slowly than those catalyzed by Xyl4, with residual starting material detectable after 1 h for all hexasaccharides.

## 4 Conclusion

Natural β‐1,3‐xylan from *C. lentillifera* contains glucose, and Xyl4 activity releases all‐β‐1,3‐linked Glc–Xyl–Xyl trisaccharide alongside Xyl–Xyl–Xyl as detected by HPLC‐MS. Using sequence‐defined synthetic glucoxylan hexasaccharides, glucose‐dependent subsite specificities and reaction patterns governing Xyl4‐catalyzed degradation were mapped. Notably, in hexasaccharide **2**, Xyl4 cleaved a β‐1,3 Glc–Xyl bond, revealing a previously unrecognized activity within GH26. These findings refine the mechanistic understanding of GH26 β‐1,3‐xylanases and motivate identification of downstream enzymes (e.g., glucosidases or xylosidases) that convert short glucoxylan oligosaccharides into monosaccharides.

To our knowledge, no bacterial species has been reported to simultaneously encode both β‐1,3‐xylanases and MLXases, reflecting ecological specialization to either green‐algal (β‐1,3‐xylan) or red‐algal (β‐1,4‐xylan and MLX) niches. Nonetheless, we show that the MLXase AlXyn26A can function as an auxiliary enzyme for converting β‐1,3‐xylan into disaccharides. Together, these results inform the selection of enzyme activities for β‐1,3‐xylan processing and point to targets for future discovery and engineering toward complete polysaccharide saccharification.

## Funding

The work was supported by European Research Council (ERC) (grant agreement no. 101087063/ASAP).

## Conflicts of Interest

The authors declare no conflicts of interest.

## Supporting information

The Supporting Information file includes ion chromatograms, replicates of enzyme‐reaction product analyses, DNS assay, general synthetic methods, automated glycan assembly procedures, and NMR spectra of synthesized oligosaccharides. References cited in the Supporting Information file are also cited in the main file.
