# Supplementary material for: Glucose Modulates Marine Xylanase Activity: Insights From Caulerpa lentillifera and Synthetic β‐1,3‐glucoxylans
Source: Chembiochem. 2026 Jul 10;27(13):e70406. doi: 10.1002/cbic.70406 (PMC13351767; doi:10.1002/cbic.70406)
Supplement: Supplementary file 1 — The Supporting Information file includes ion chromatograms, replicates of enzyme‐reaction product analyses, DNS assay, general synthetic methods, automated glycan assembly procedures, and NMR spectra of synthesized oligosaccharides. References cited in the Supporting Information file are also cited in the main file. [file CBIC-27-e70406-s001.pdf]

Supporting Information

For

Glucose Modulates Marine Xylanase Activity: Insights from *Caulerpa lentillifera* and Synthetic β-1,3-glucoxylans

Nils H. Rustmeier, Nitish Verma, and Fabian Pfrengle*

BOKU University

Department of Natural Sciences and Sustainable Resources, Institute of Organic Chemistry

Muthgasse 18, 1190 Vienna, Austria

*E-mail: fabian.pfrengle@boku.ac.at

Table of Contents

1. Ion chromatograms from endpoint HPLC-MS analyses of the xylanase reaction products 1

2. HPLC-MS analyses of enzymatic reaction products from synthetic glucoxylan oligosaccharide substrates (technical duplicates) 4

3. Determination of specific xylanase activities 4

4. General methods for synthetic procedures 5

4.1 General method for analytical HPLC of crude reaction mixtures obtained after photocleavage 5

Table S2. Analytical and semi-prep HPLC columns with their respective specifications. 5

Table S3. Analytical HPLC method (50 min) for YMC-Small normal phase (NP) column. 5

4.2 Method for calculation of %yield of purified glucoxylan hexasaccharides after global deprotection 6

5. Automated Glycan Assembly (AGA) of glucoxylan hexasaccharides 7

5.1 AGA of glucoxylan hexasaccharide **1** 7

5.2 AGA of glucoxylan hexasaccharide **2** 18

5.3 AGA of glucoxylan hexasaccharide **3** 28

Table S4. NMR chemical shifts (ppm) of selected proton and carbon atoms in glucoxylan hexasaccharides **1–3** 39

5. References 39

1. Ion chromatograms from endpoint HPLC-MS analyses of the xylanase reaction products

No-enzyme control, 0.1% (w/v) β-1,3-xylan

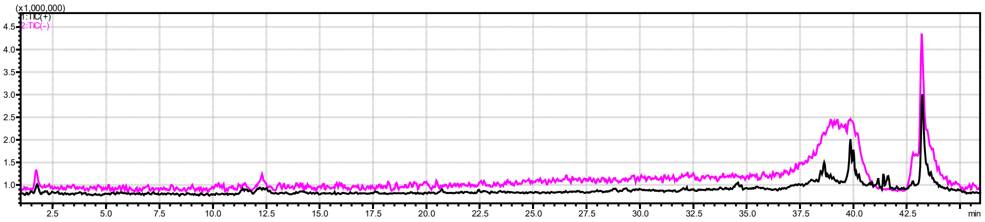

1 μM Xyl4, 0.1% (w/v) β-1,3-xylan, 24 h

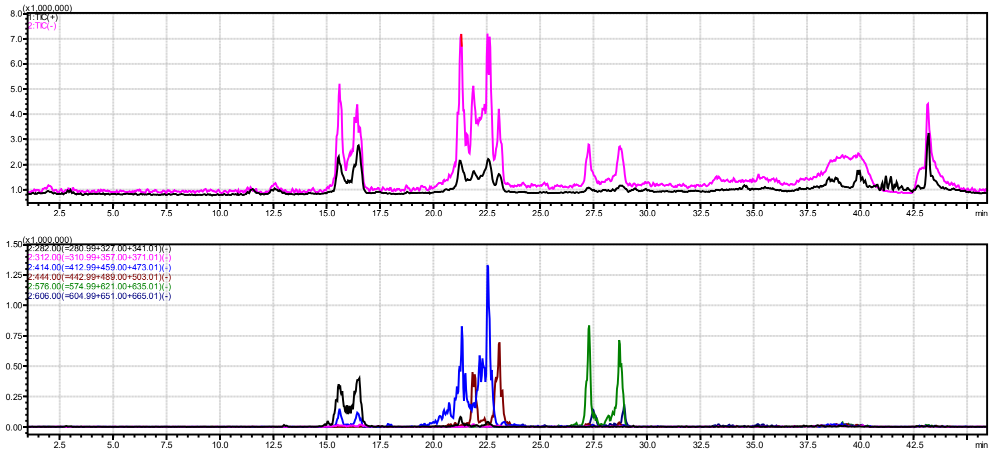

1 μM AXyn26A, 0.1% (w/v) β-1,3-xylan, 24 h

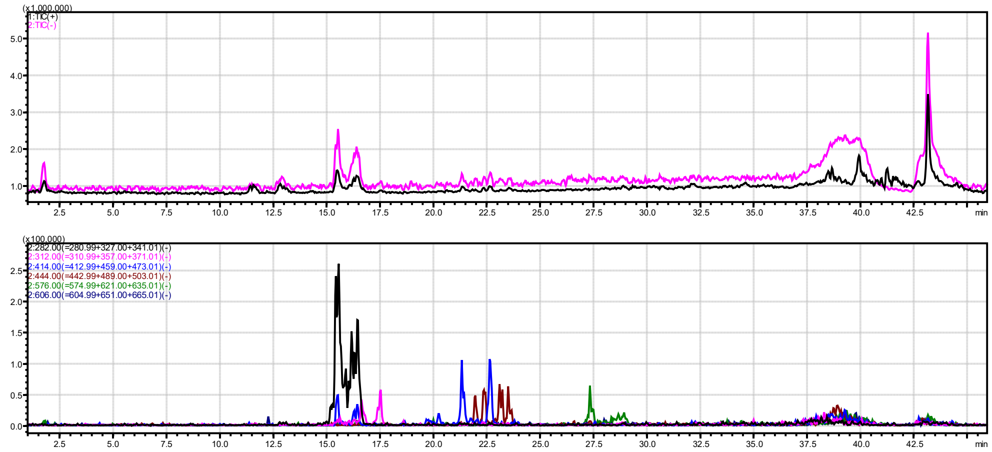

1 μM Xyl4 + 1 μM AXyn26A, 0.1% (w/v) β-1,3-xylan, 24 h

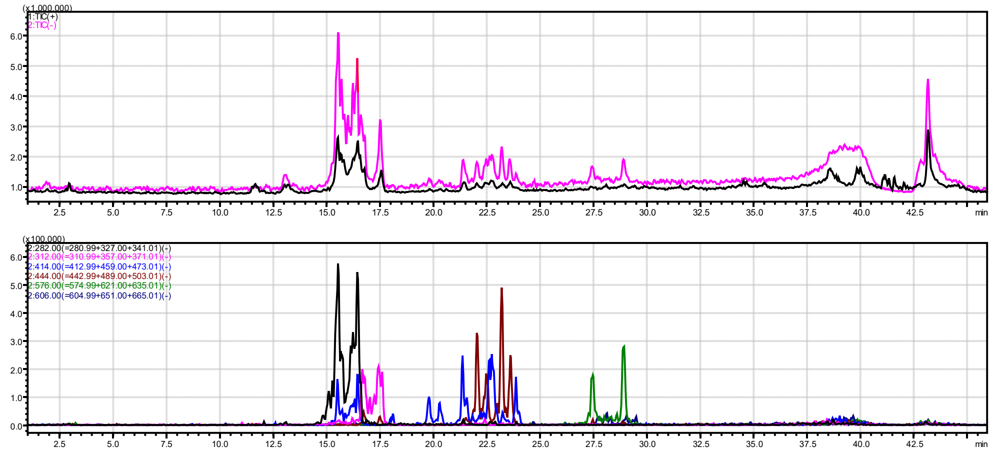

Figure S1. Total ion chromatograms in positive (+) and negative (−) modes, together with extracted ion chromatograms for native and linker-functionalized (gluco-)xylan oligosaccharides, plotted as ion counts versus retention time (min). The extracted m/z values (with one glucose per species) are m/z = 282/321 for disaccharides, 414/444 for trisaccharides, and 576/606 for tetrasaccharides. Pentasaccharides (not extracted) elute at ~35 min, as observed in the TICs.

1 μM Xyl4, 0.1% (w v) hexasaccharide **1**, 24 h

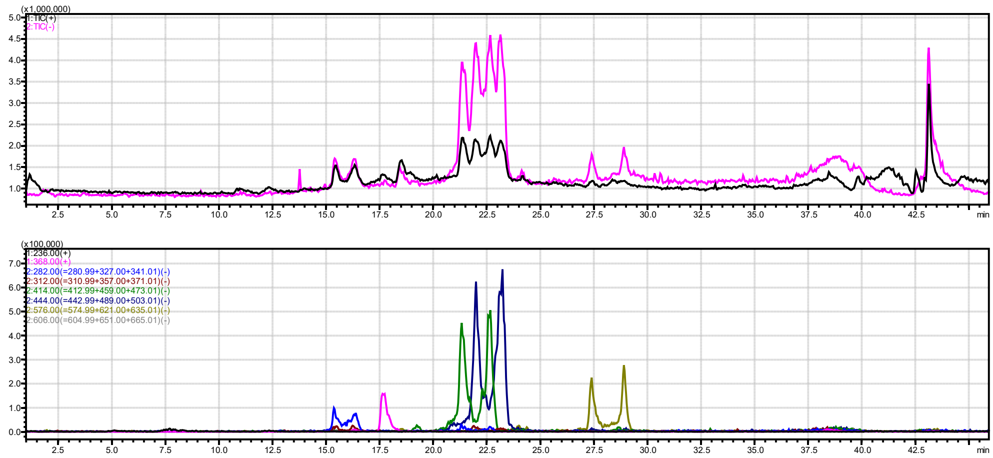

1 μM Xyl4, 0.1% (w v) hexasaccharide **2**, 24 h

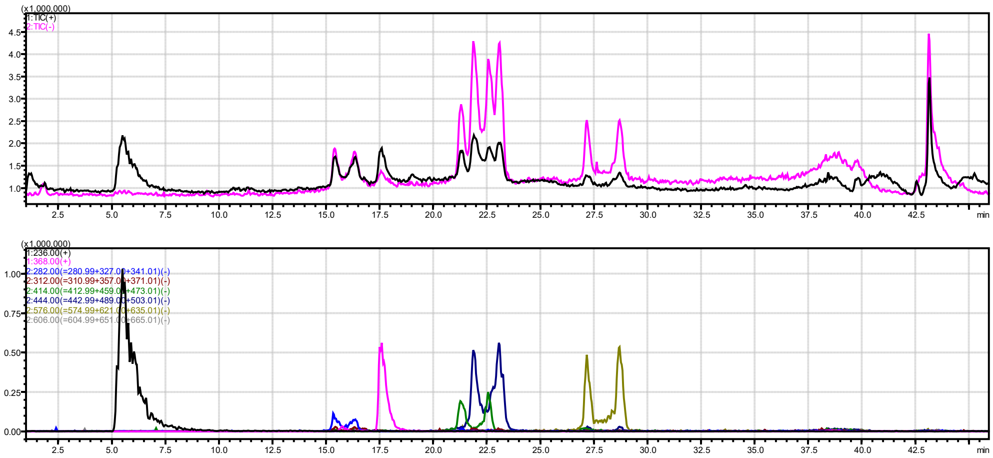

1 μM Xyl4, 0.1% (w v) hexasaccharide **3**, 24 h

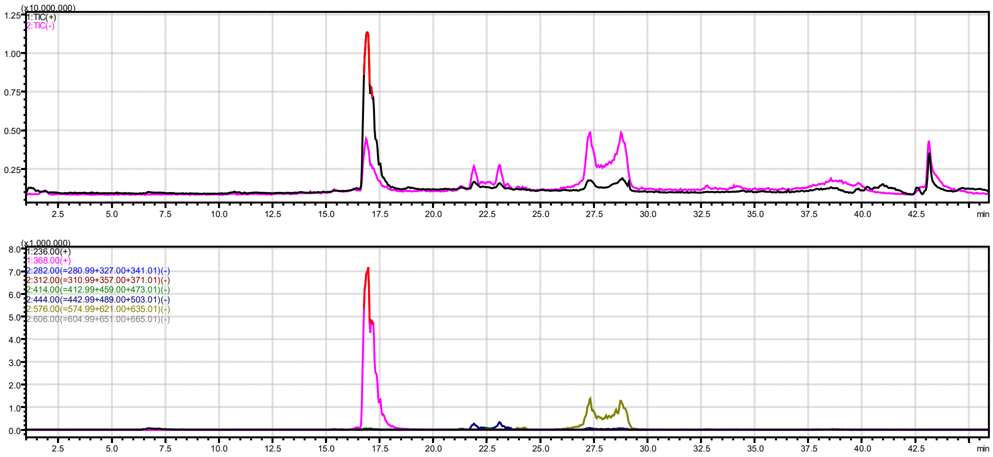

Figure S1. *continued*

1 μM AXyn26A, 0.1% (w v) hexasaccharide **1**, 24 h

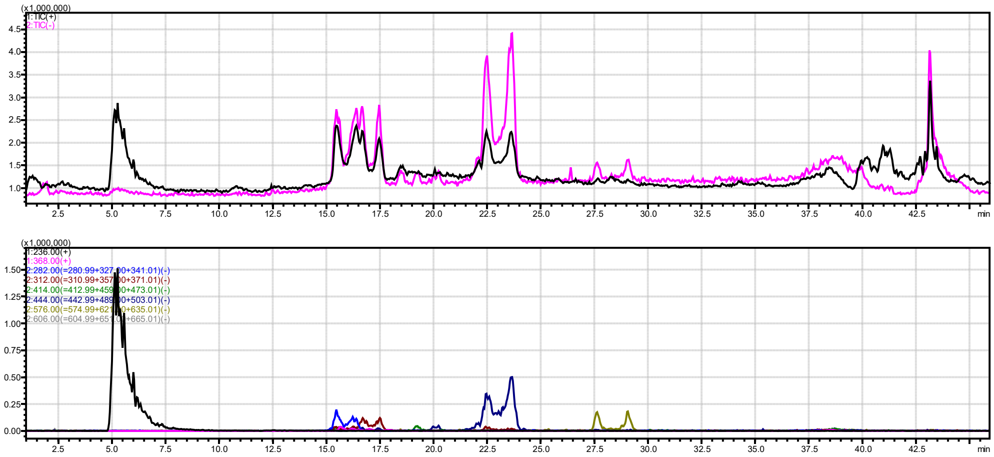

1 μM AXyn26A, 0.1% (w v) hexasaccharide **2**, 24 h

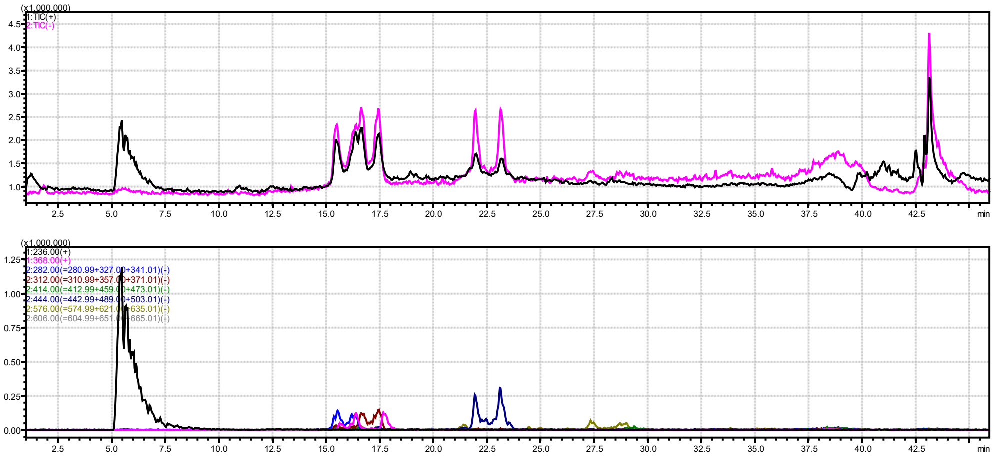

1 μM AXyn26A, 0.1% (w v) hexasaccharide **3**, 24 h

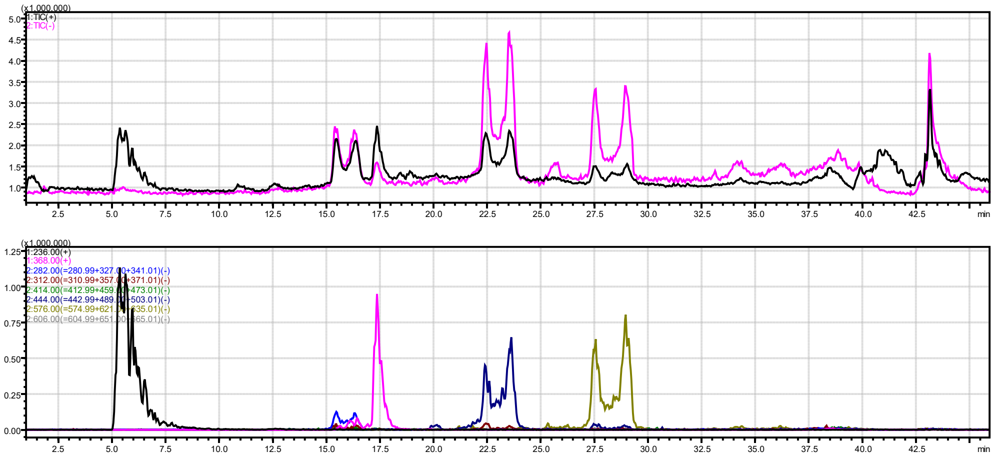

Figure S1. *continued*

2. HPLC-MS analyses of enzymatic reaction products from synthetic glucoxylan oligosaccharide substrates (technical duplicates)

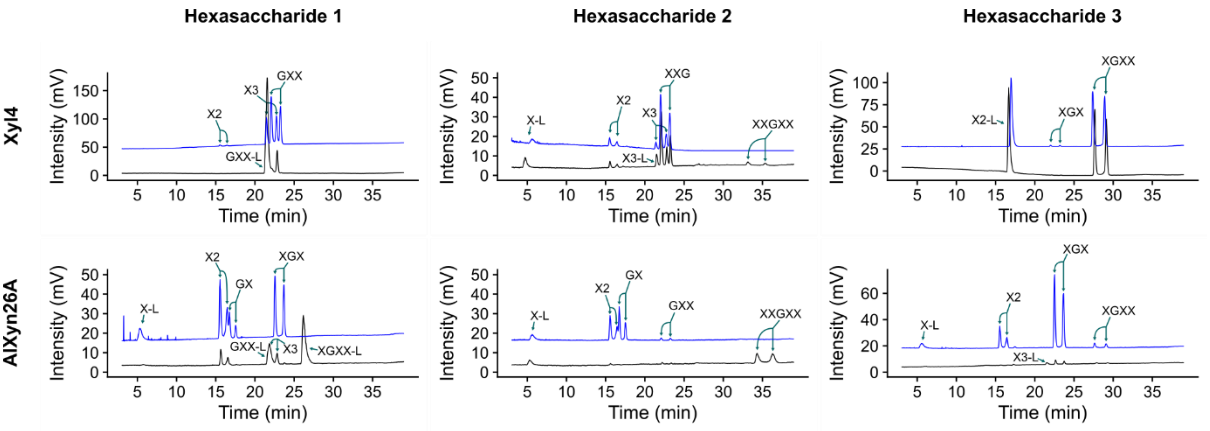

Figure S2. Technical duplicate HPLC-MS analyses of synthetic β-1,3-glucoxylan oligosaccharide digests. Result panels are arranged in a grid with substrates at the top and enzymes shown on the left. Plots show ELSD intensities (mV) vs. retention time (min), with product annotations as in main file Figure 2.

3. Determination of specific xylanase activities

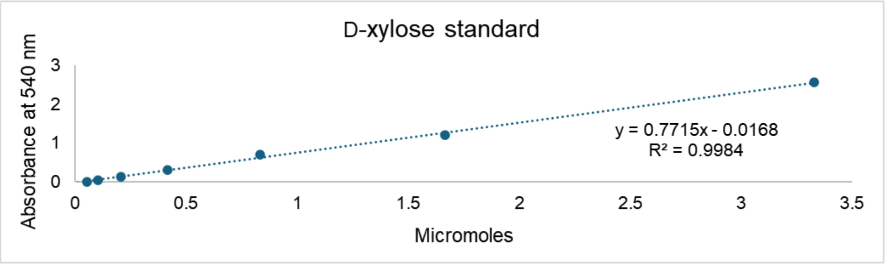

Figure S3. Photometric calibration of D-xylose at 540 nm.

Table S1. Conversion of absorbances from DNS assay to specific xylanase activities. Xylose-equivalents (µmol) were calculated using the rearranged linear equation from Fig. S3, $x = (y + 0.0168) / 0.7715$, where y is the absorbance at 540 nm (Abs_{540}). Following controls subtraction, specific activities were derived as U = µmol min^{-1} per mg of enzyme present. Standard deviations (SD) were propagated by quadrature (square root of summed squares).

Enzyme	Molarity (µM)	MW (g/mol)	Mass concentration (mg/mL)	Total mass (mg)
AlXyn26A	7.8	36970.55	0.288	0.029
Xyl4	1.67	37280.79	0.062	0.006

**Samples**	**n**	**Abs_{540}**	**Xylose-equivalents (µmol)**	**µmol min^{-1} (U)**	**Average_U**	**SD_U**	**Specific activity (U/mg)**	**± SD**
	1	0.554	0.739	0.074				
MLX controls	2	0.504	0.675	0.068	0.068	0.005		
	3	0.470	0.630	0.063				
	1	1.020	1.344	0.134				
MLX + AlXyn26A	2	1.027	1.353	0.135	0.136	0.002	**2.355**	**0.204**
	3	1.052	1.385	0.139				
	1	0.018	0.045	0.005				
β-1,3-xylan controls	2	0.007	0.031	0.003	0.004	0.001		
	3	0.010	0.035	0.003				
	1	0.084	0.131	0.013				
β-1,3-xylan + Xyl4	2	0.150	0.216	0.022	0.015	0.006	**1.846**	**0.920**
	3	0.067	0.109	0.011				

4. General methods for synthetic procedures

4.1 General method for analytical HPLC of crude reaction mixtures obtained after photocleavage

The obtained crude reaction mixture in DCM after the photocleavage reaction was concentrated under reduced pressure, redissolved in EtOAc, and passed through a pre-packed 0.5 g normal phase silica column (SI-S-500/6). Eluted EtOAc-fractions were concentrated under reduced pressure, and the crude mixture was re-dissolved in 3 mL toluene (unless stated otherwise) under sonication. The crude reaction mixture in toluene (70 µL) was then injected into the analytical HPLC-column.

Table S2. Analytical and semi-prep HPLC columns with their respective specifications.

	Column type	Column specifications
**Analytical HPLC**		
1	YMC-Small	YMC-Pack SIL-06 (250 × 4.6 mmI.D., S-5 µm, 6 nm)
**Semi-prep HPLC (for purification)**		
2	YMC-Small	YMC-Pack SIL-06 (250 × 10.0 mmI.D., S-5 µm, 6 nm)

Table S3. Analytical HPLC method (50 min) for YMC-Small normal phase (NP) column.

Time [min]	A (EtOAc%)	B (hexanes%)	Flow (mL/min)	Max. Pressure Limit [bar]
0	20	80	1	400.00
5	20	80	1	…..
35	50	50	1	…..
40	55	45	1	…..
45	55	45	1	…..
50	20	80	1	…..

4.2 Method for calculation of %yield of purified glucoxylan hexasaccharides after global deprotection

Unprotected glucoxylan hexasaccharides were isolated in the salt form with acetate as a counter ion. Yield of unprotected hexasaccharides were precisely calculated by determining the amount of acetate present in the NMR sample. Please see the following standard equations for calculating the yield.

$Wt._A$ + $Wt._B$ = x mg (known weight after the lyophilization)

$n_A/n_B = 1/(I_B/3) = y$ (known number from the ^{1}H NMR spectrum)

$n_B = n_A/y$

In general, n = Wt./M.Wt.

therefore, Wt. = n × M.Wt.

$n_A \times M.Wt._A + n_B \times M.Wt._B = x$ mg

$n_A \times M.Wt._A + (n_A/y) \times M.Wt._B = x$ mg

By solving the above equation, the defined value of n_A was calculated.

$$\%\ \text{yield} = \frac{\text{nA}}{\text{nSM}}\ \text{X}\ 100$$

$Wt._A$ = weight of the desired hexasaccharide with linker in free NH_2 form

$M.Wt._A$ = molecular weight of the desired hexasaccharide with linker in free NH_2 form

n_A = moles of the desired hexasaccharide with linker in free NH_2 form

$Wt._B$ = weight of the AcOH

$M.Wt._B$ = molecular weight of the AcOH = 60.052 g/mol

n_B = moles of the AcOH

I_B = integration value of the CH_3 of the AcOH at ~1.89 ppm in the ^{1}H NMR spectrum

nSM = no. of moles of the starting material (protected hexasaccharide)

5. Automated Glycan Assembly (AGA) of glucoxylan hexasaccharides

5.1 AGA of glucoxylan hexasaccharide 1

Benzyloxycarbonylaminopentyl 2-*O*-benzoyl-4-*O*-benzyl-β-D-xylopyranosyl-(1→3)-2-*O*-benzoyl-4-*O*-benzyl-β-D-xylopyranosyl-(1→3)-2-*O*-benzoyl-4-*O*-benzyl-β-D-xylopyranosyl-(1→3)-2-*O*-benzoyl-4,6-di-*O*-benzyl-β-D-glucopyranosyl-(1→3)-2-*O*-benzoyl-4-*O*-benzyl-β-D-xylopyranosyl-(1→3)-2-*O*-benzoyl-4-*O*-benzyl-β-D-xylopyranoside (S1)

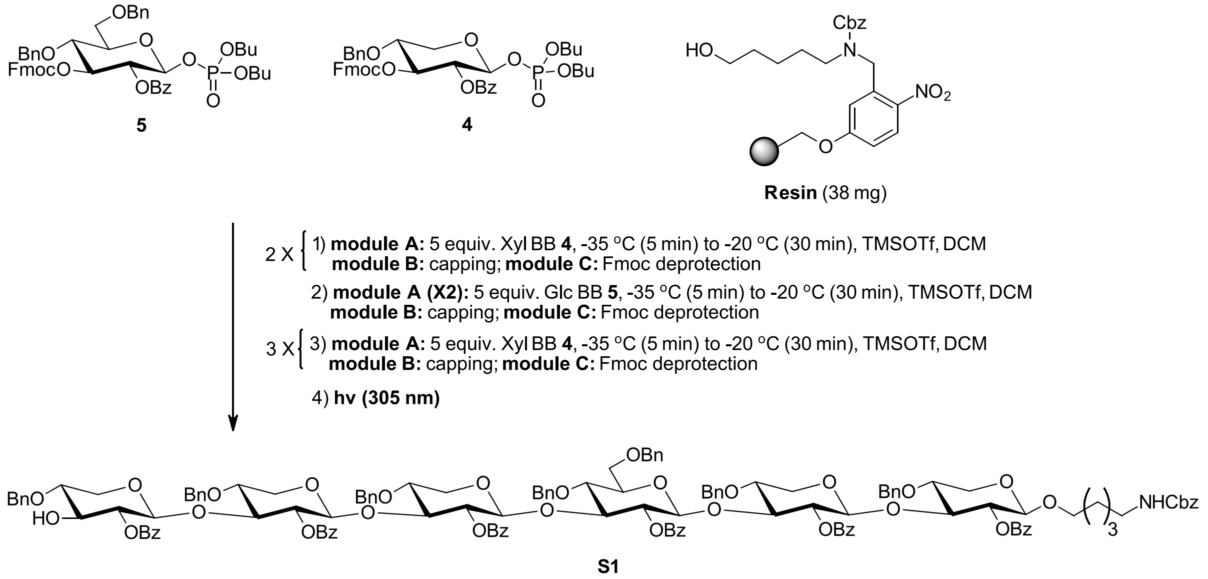

Experimental procedure: Linker-functionalized resin (38 mg, 12.5 µmol) was placed in the synthesizer and synthesizer modules were applied as follows:

2 × [

1) module A (Xylose BB **4** [1], 47 mg, 0.0625 mmol, 5 equiv.) at –35ºC (5 min) to –20ºC (30 min), module B, and module C
   ]
2) two cycles of module A (Glucose BB **5** [2], 55 mg, 0.0625 mmol, 5 equiv.) at –35ºC (5 min) to –20ºC (30 min), module B, and module C

3 × [

3) module A (Xylose BB **4**, 47 mg, 0.0625 mmol, 5 equiv.) at –35ºC (5 min) to –20ºC (30 min), module B, and module C
   ].

Cleavage from the resin using UV irradiation at 305 nm in a continuous flow photoreactor afforded the crude product. Purification of the crude by normal phase HPLC using a preparative YMC-Small column (EtOAc/hexanes = 1/2.3 to 1/1, v/v) gave protected glucoxylan hexasaccharide **S1** (12.3 mg, 43% yield over 13 steps) as a glassy solid.

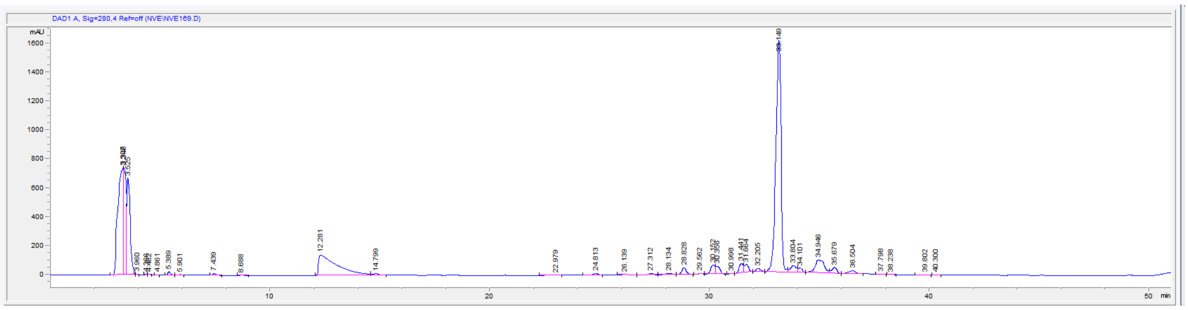

Analytical HPLC of the crude reaction mixture was performed on a YMC-Small NP column using a gradient of EtOAc in hexanes (50 min, flow rate 1 mL/min).

^{1}H NMR (600 MHz, $(CD_3)_2CO$): *δ* 7.89-7.82 (m, 6H, Ar-H), 7.70-7.66 (m, 2H, Ar-H), 7.63-7.59 (m, 4H, Ar-H), 7.58-7.49 (m, 3H, Ar-H), 7.48-7.43 (m, 4H, Ar-H), 7.43-7.38 (m, 2H, Ar-H), 7.37-7.34 (m, 11H, Ar-H), 7.33-7.26 (m, 28H, Ar-H), 7.25-7.19 (m, 10H, Ar-H), 6.09 (br, 1H, N-H), 5.03 (s, 2H, CH_2-Cbz), 4.98 (dd, *J* = 7.4, 9.0 Hz, 1H, H-2F), 4.91-4.85 (m, 2H, H-2C, CH_2-Ph), 4.84 (dd, *J* = 5.2, 6.4 Hz, 1H, H-2E), 4.82-4.79 (m, 2H, H-1C, H-1B), 4.78-4.56 (m, 14H, H-1F, H-2B, 7H's of CH_2-Ph, H-2D, H-2A, H-1E, H-1D, O-H), 4.53-4.45 (m, 6H, CH_2-Ph), 4.42 (d, *J* = 7.2 Hz, 1H, H-1A), 4.05-3.96 (m, 6H, H-5B, H-3B, H-5F, H-5E, H-3C, H-3E), 3.90 (t, *J* = 8.3 Hz, 1H, H-3A), 3.86-3.79 (m, 2H, H-5D, H-5A), 3.71-3.64 (m, 3H, H-3D, H-6C, H-3F), 3.63-3.52 (m, 3H, OCH_2, H-6C, H-4F), 3.51-3.44 (m, 3H, H-5C, H-4E, H-4B), 3.34-3.26 (m, 3H, H-4C, OCH_2, H-5B), 3.25-3.16 (m, 5H, H-4A, H-5E, H-5A, H-4D, H-5F), 3.096 (dd, *J* = 6.0, 12.0 Hz, 1H, H-5D), 2.94-2.88 (m, 2H, CH_2-NHCbz), 1.40-1.31 (m, 4H, CH_2), 1.18-1.11 (m, 2H, CH_2) ppm.

^{13}C NMR (151 MHz, $(CD_3)_2CO$): *δ* 165.98, 165.18, 165.14, 165.05, 157.03, 139.91, 139.75, 139.71, 139.69, 139.67, 134.07, 133.86, 133.71, 133.65, 133.62, 131.29, 130.92, 130.69, 130.63, 130.60, 130.58, 130.54, 130.52, 130.48, 130.27, 129.44, 129.18, 129.15, 129.10, 129.05, 129.03, 129.00, 128.97, 128.95, 128.91, 128.77, 128.74, 128.62, 128.60, 128.55, 128.51, 128.37, 128.27, 128.23, 128.21, 128.19, 128.12, 128.06, 101.65, 101.45, 100.87, 100.71, 100.20, 99.76, 79.92, 78.58, 78.55, 78.23, 76.97, 76.80, 76.76, 76.28, 76.17, 76.01, 75.69, 75.51, 75.33, 75.17, 74.92, 74.89, 74.86, 74.82, 74.72, 74.38, 73.75, 73.61, 73.40, 73.25, 72.60, 72.37, 72.33, 71.87, 70.04, 69.30, 66.26, 64.42, 63.90, 63.13, 61.99, 61.29, 41.34, 30.22 (CH_2-linker, merged with solvent peak), 29.77 (CH_2-linker, merged with solvent peak), 23.75 ppm.

ESI-HRMS: m/z $[M + NH_4]^+$ calcd. for $C_{135}H_{139}N_2O_{34}$: 2331.9204; found 2331.9239.

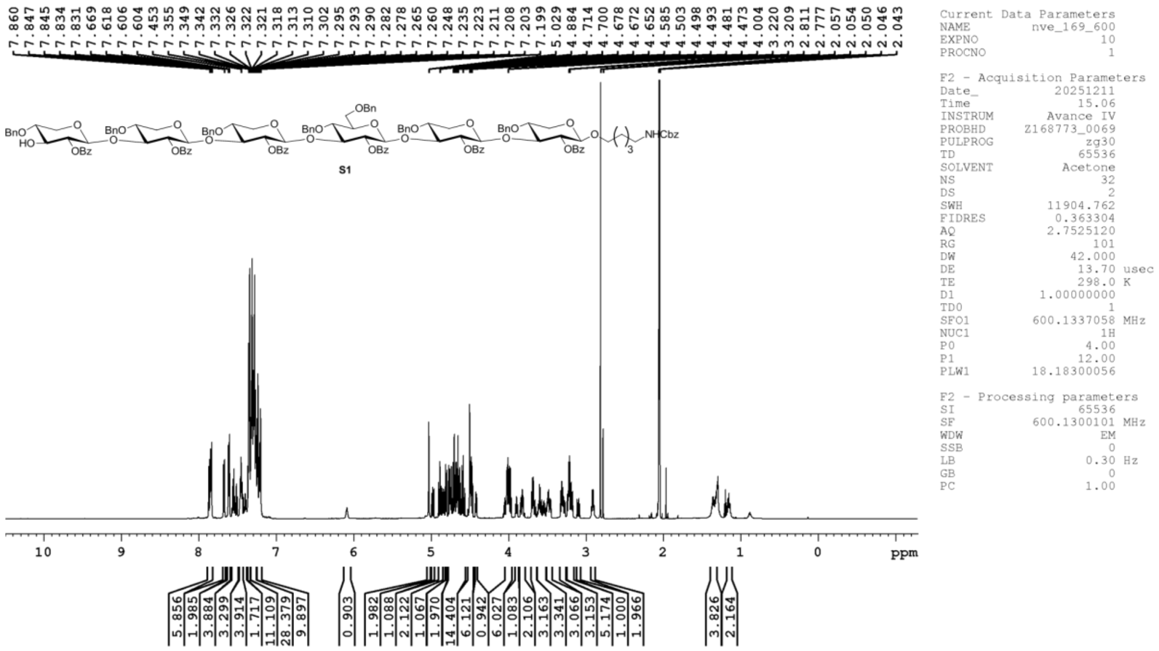

^{1}H NMR spectrum of compound **S1** (600 MHz, $(CD_3)_2CO$)

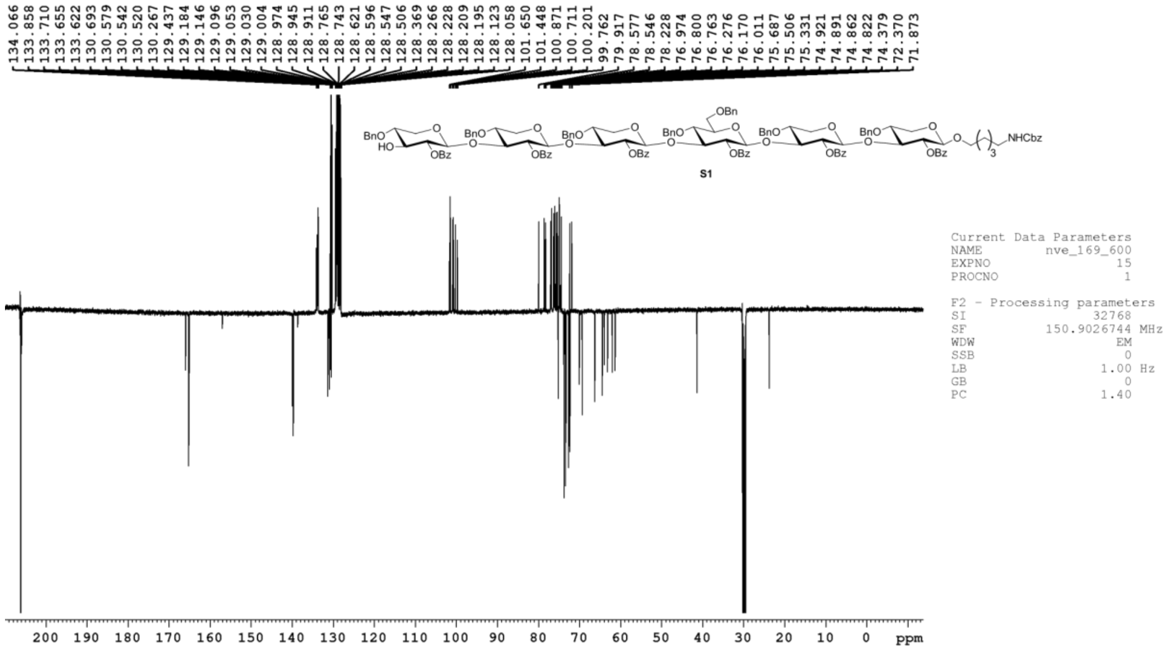

^{13}C APT NMR spectrum of compound **S1** (151 MHz, $(CD_3)_2CO$)

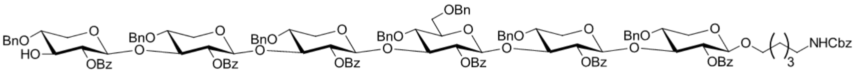

S1

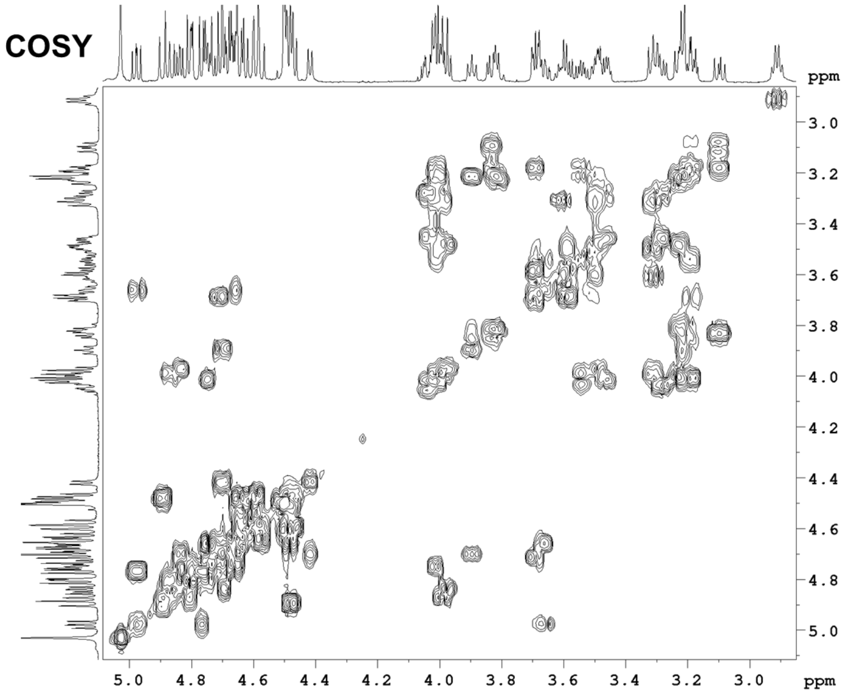

^{1}H-^{1}H COSY NMR spectrum of compound **S1** (600 MHz, $(CD_3)_2CO$)

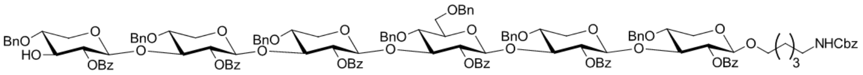

S1

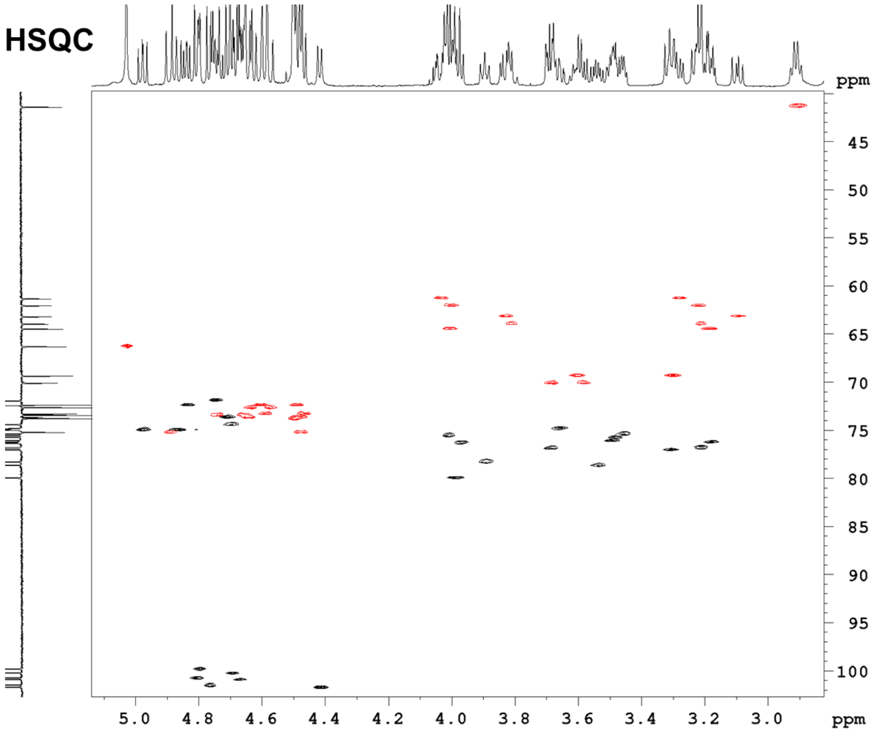

^{1}H-^{13}C HSQC NMR spectrum of compound **S1** (600/151 MHz, $(CD_3)_2CO$)

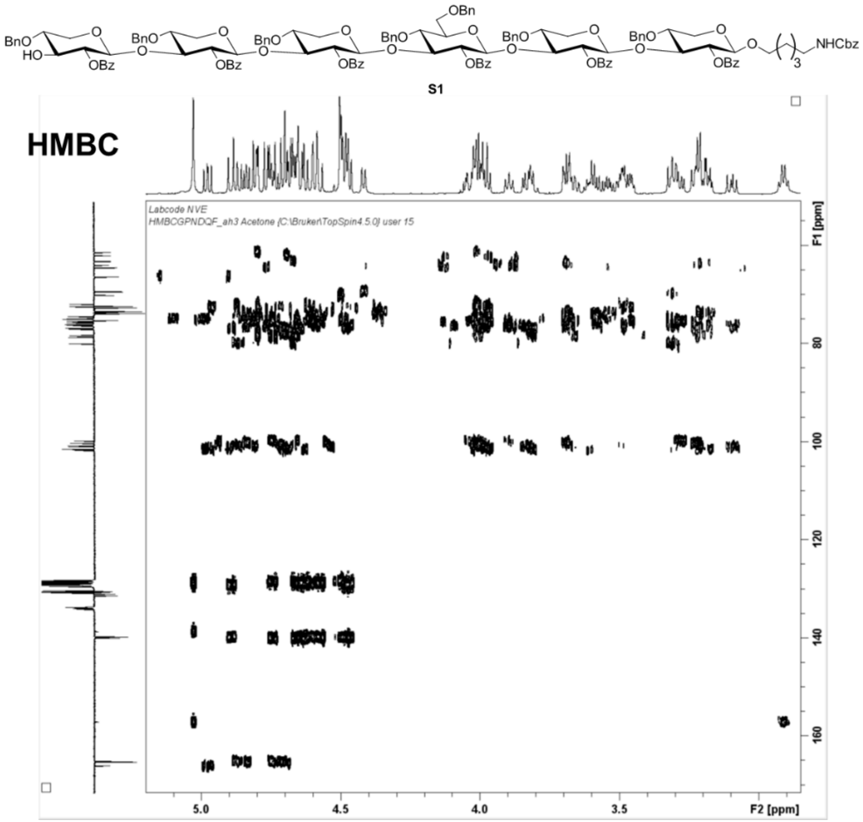

^{1}H-^{13}C HMBC NMR spectrum of compound **S1** (600/151 MHz, $(CD_3)_2CO$)

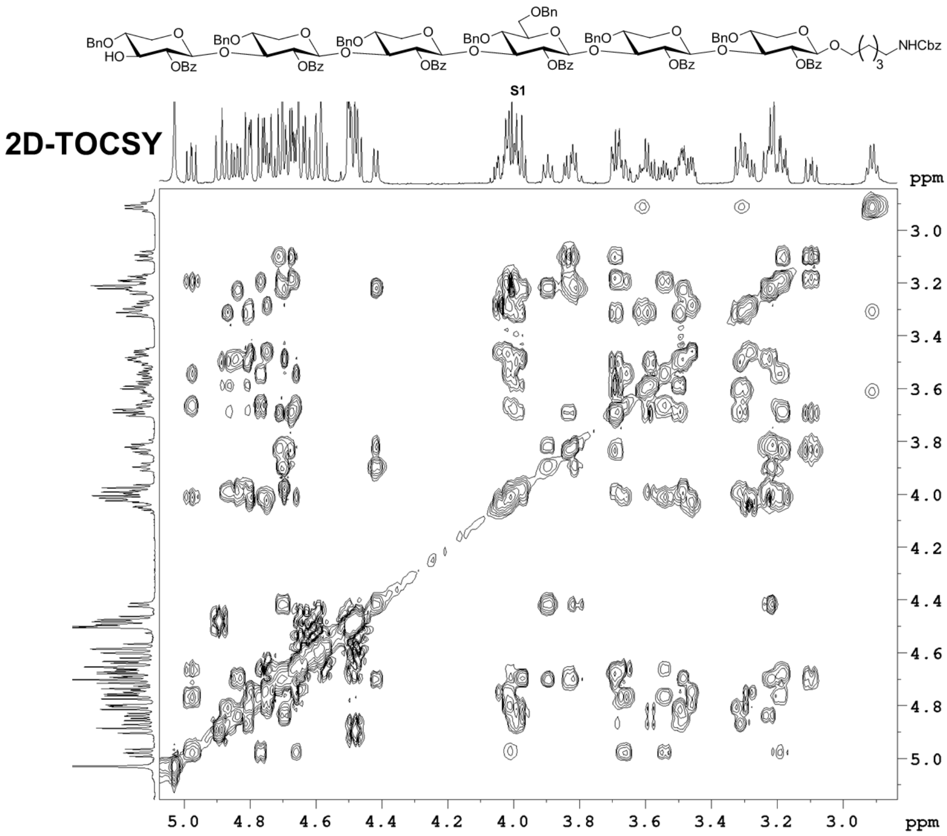

^{1}H-^{1}H TOCSY NMR spectrum of compound **S1** (600 MHz, $(CD_3)_2CO$)

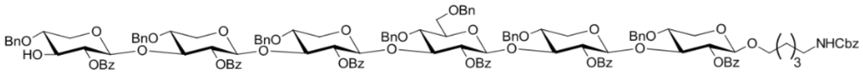

S1

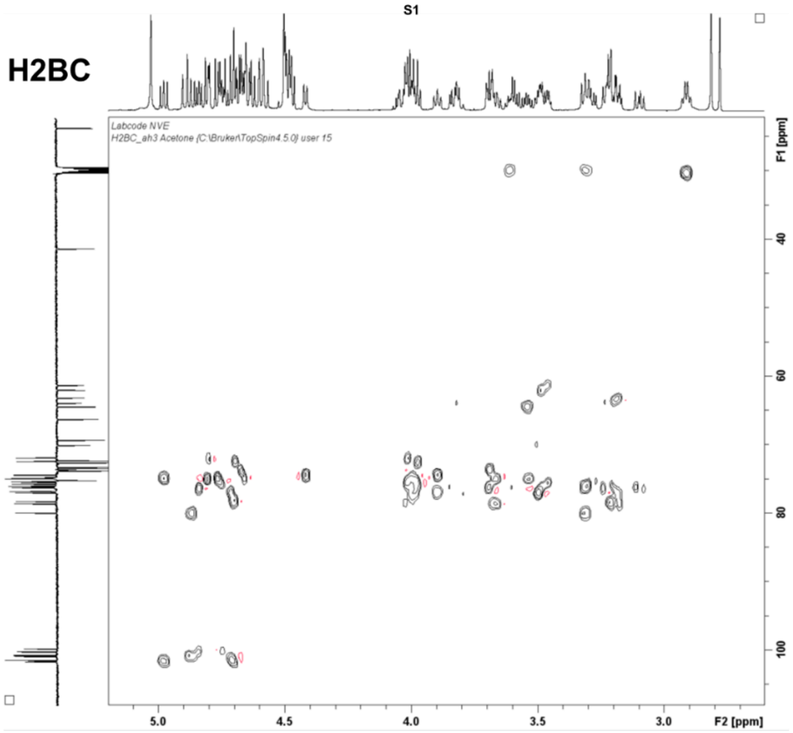

^{1}H-^{13}C H2BC NMR spectrum of compound **S1** (600/151 MHz, $(CD_3)_2CO$)

Aminopentyl β-D-xylopyranosyl-(1→3)-β-D-xylopyranosyl-(1→3)-β-D-xylopyranosyl-(1→3)-β-D-glucopyranosyl-(1→3)-β-D-xylopyranosyl-(1→3)-β-D-xylopyranoside (1)

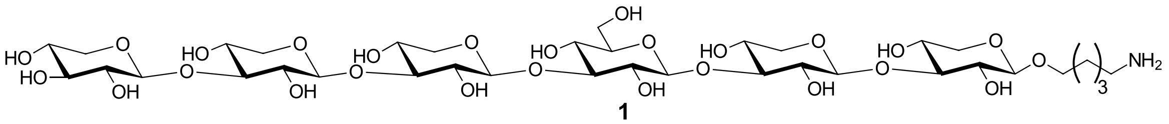

1

Experimental procedure: To a solution of protected hexasaccharide **S1** (12.3 mg, 5.31 µmol) in THF (319 µL, 17 mM), a solution of NaOMe in MeOH (0.5 M, 159 µL, 79.7 µmol) was added at rt, and the reaction mixture was allowed to stir overnight. After confirmation of reaction completion (23 h) by TLC (R_f: 0.53 (MeOH/$CHCl_3$ = 1/15, v/v)), the reaction mixture was neutralized by the addition of IR-120 H^+ resin while slowly stirring the mixture. Then, the reaction mixture was filtered, and the filtrate was concentrated under reduced pressure to yield a partially deprotected glassy crude, which was kept under high vacuum until usage in the next step without any further purification. To a solution of partially deprotected crude (5.31 µmol) in *t*-BuOH (800 µL), H_2O (400 µL), and AcOH (200 µL), unreduced 10% Pd/C (17.1 mg) was added, and the reaction mixture was stirred in the H_2 reactor under a pressure of 8 bar H_2. After 72 h, the reaction mixture was filtered using a PTFE syringe filter (0.45 µm) and concentrated under reduced pressure to yield a crude product, which was purified using pre-packed C18 (500 mg, 6 mL) column chromatography with H_2O/ACN (100% to 80%, v/v, + 0.1% AcOH). The purified product was lyophilized to give glucoxylan hexasaccharide **1** (3.94 mg, 74% yield over 2 steps) as a white amorphous foam.

^{1}H NMR (600 MHz, D_2O): δ 4.76 (1H, merged with D_2O peak, H-1C), 4.72-4.68 (m, 3H, H-1D, H-1E, H-1F), 4.66 (d, J = 7.8 Hz, 1H, H-1B), 4.41 (d, J = 7.9 Hz, 1H, H-1A), 4.03-3.94 (m, 5H, H-5A, H-5B, H-5D, H-5E, H-5F), 3.92-3.84 (m, 2H, H-6C, OCH_2), 3.75-3.59 (m, 12H, H-6C, OCH_2, H-4A, H-3A, H-4B), 3.55-3.40 (m, 8H, H-2C, H-5C, H-3B, H-2A, H-2D, H-2E, H-2F), 3.35-3.26 (m, 6H, H-2B, H-5A, H-5B, H-5D, H-5E, H-5F), 2.99 (*t*, J = 7.6 Hz, 2H, C*H*$_2$-NH_2), 1.71-1.61 (m, 4H, CH_2), 1.47-1.40 (m, 2H, CH_2) ppm.

^{13}C NMR (151 MHz, D_2O): δ 104.04, 103.85, 103.81, 103.78, 103.30, 103.04, 84.40, 84.38, 84.24, 84.08, 76.31, 76.22, 73.97, 73.93, 73.68, 73.65, 73.33, 70.76, 69.80, 68.62, 68.42, 68.28, 68.26, 65.77, 65.41, 65.34, 61.31, 39.95, 28.79, 26.99, 22.70 ppm.

ESI-HRMS: m/z [M + Na]$^+$ calcd. for $C_{36}H_{63}NO_{26}Na$: 948.3531; found 948.3533.

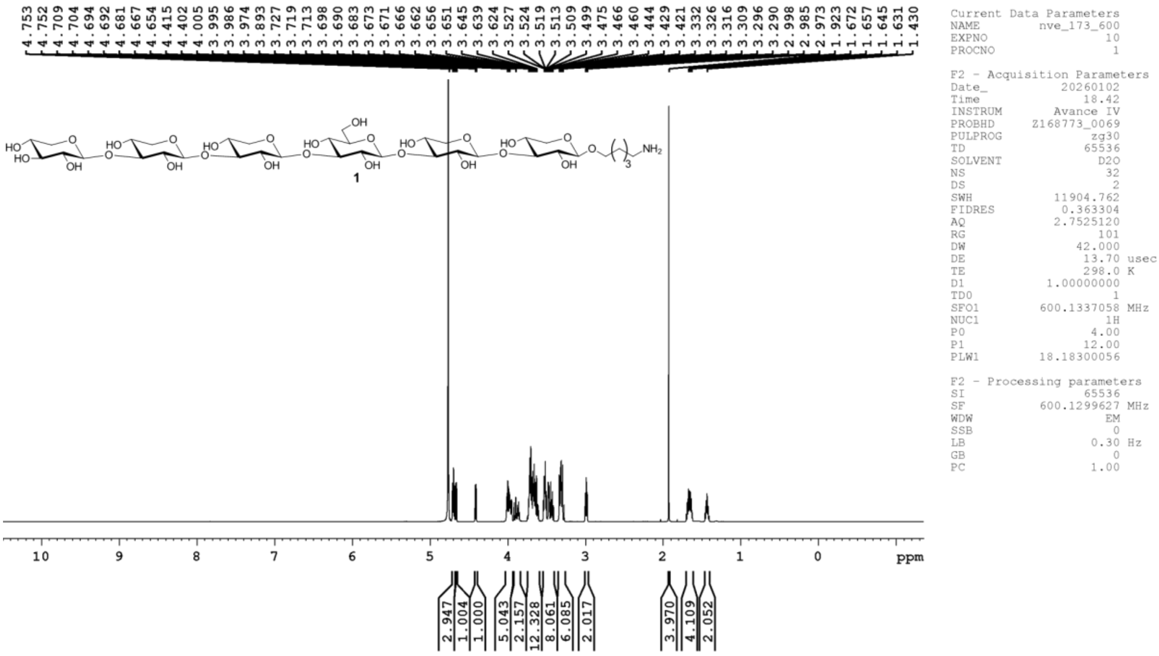

^{1}H NMR spectrum of compound **1** (600 MHz, D_2O)

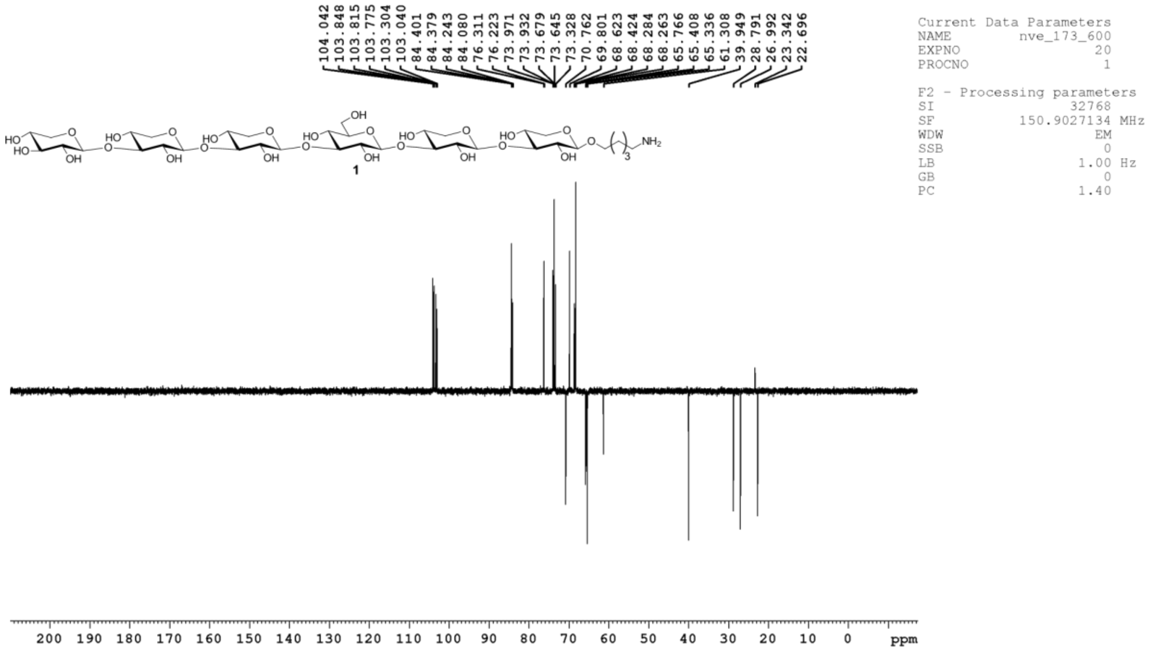

^{13}C APT NMR spectrum of compound **1** (151 MHz, D_2O)

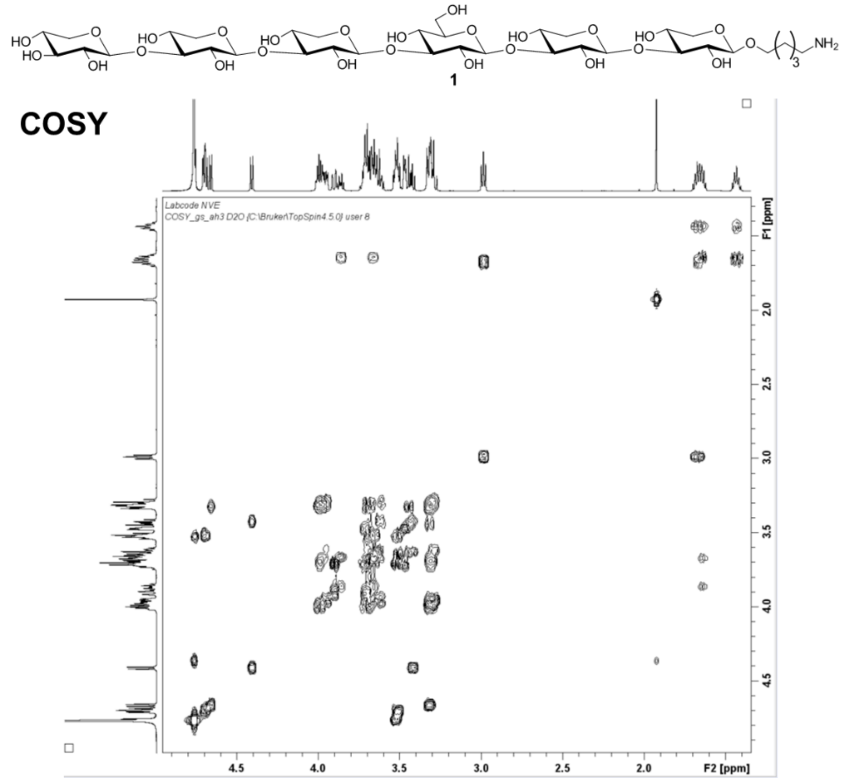

^{1}H-^{1}H COSY NMR spectrum of compound **1** (600 MHz, D_2O)

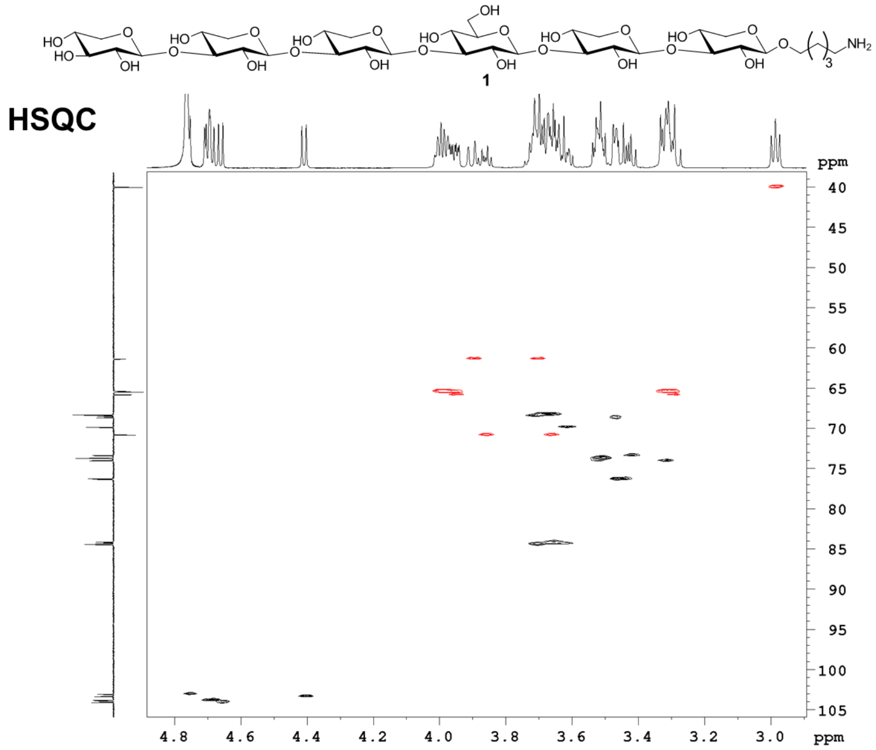

^{1}H-^{13}C HSQC NMR spectrum of compound **1** (600/151 MHz, D_2O)

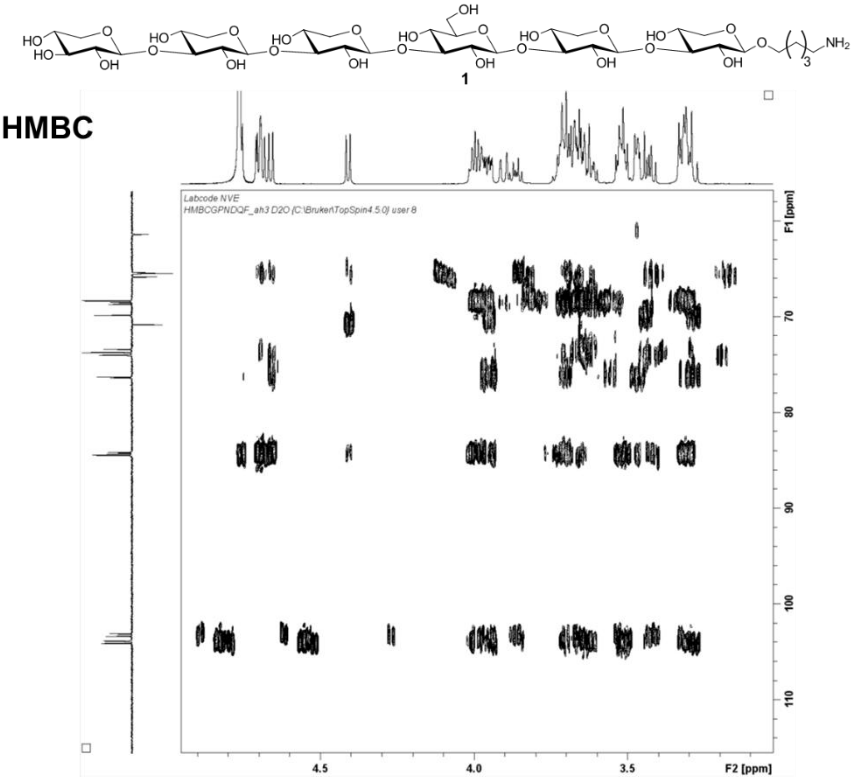

^{1}H-^{13}C HMBC NMR spectrum of compound **1** (600/151 MHz, D_2O)

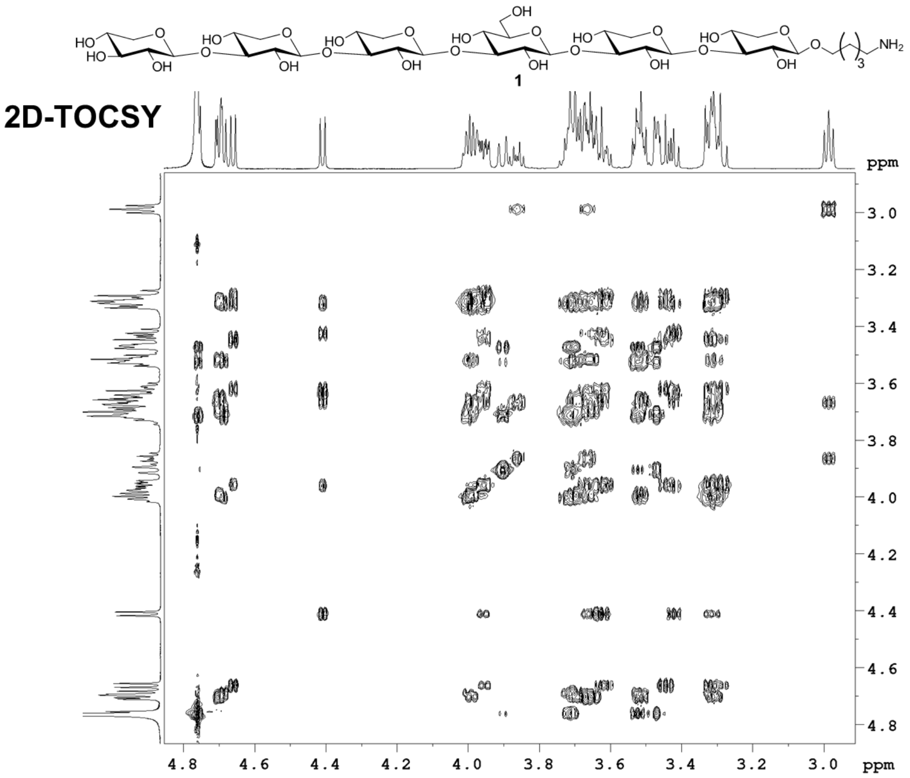

^{1}H-^{1}H TOCSY NMR spectrum of compound **1** (600 MHz, D_2O)

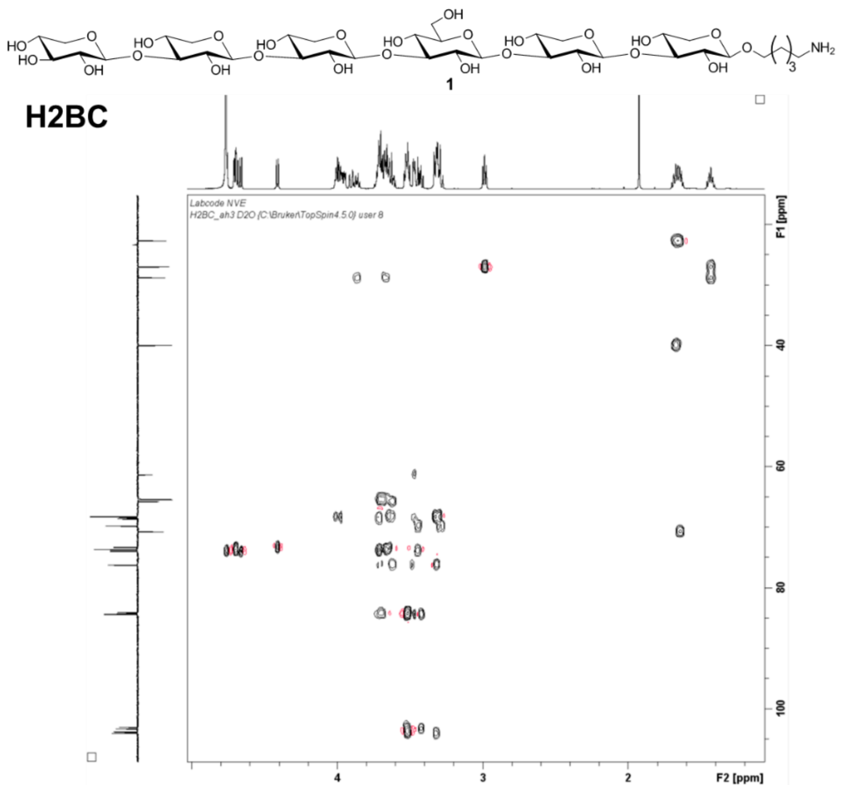

^{1}H-^{13}C H2BC NMR spectrum of compound **1** (600/151 MHz, D_2O)

5.2 AGA of glucoxylan hexasaccharide 2

Benzyloxycarbonylaminopentyl 2-*O*-benzoyl-4-*O*-benzyl-β-D-xylopyranosyl-(1→3)-2-*O*-benzoyl-4-*O*-benzyl-β-D-xylopyranosyl-(1→3)-2-*O*-benzoyl-4,6-di-*O*-benzyl-β-D-glucopyranosyl-(1→3)-2-*O*-benzoyl-4-*O*-benzyl-β-D-xylopyranosyl-(1→3)-2-*O*-benzoyl-4-*O*-benzyl-β-D-xylopyranosyl-(1→3)-2-*O*-benzoyl-4-*O*-benzyl-β-D-xylopyranoside (S2)

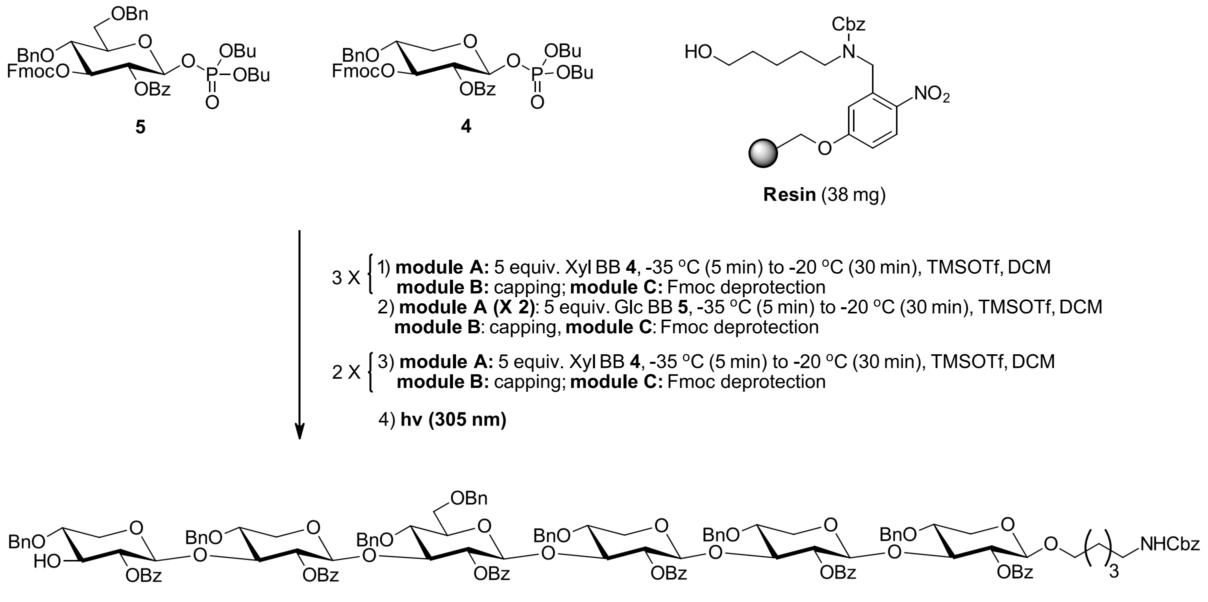

S2

Experimental procedure: Linker-functionalized resin (38 mg, 12.5 µmol) was placed in the synthesizer and synthesizer modules were applied as follows:

3 × [

1) module A (Xylose BB **4**, 47 mg, 0.0625 mmol, 5 equiv.) at –35°C (5 min) to –20°C (30 min), module B, and module C
]
2) two cycles of module A (Glucose BB **5**, 55 mg, 0.0625 mmol, 5 equiv.) at –35°C (5 min) to –20°C (30 min), module B, and module C

2 × [

3) module A (Xylose BB **4**, 47 mg, 0.0625 mmol, 5 equiv.) at –35°C (5 min) to –20°C (30 min), module B, and module C
].

Cleavage from the resin using UV irradiation at 305 nm in a continuous flow photoreactor afforded the crude product. Purification of the crude by normal phase HPLC using a preparative YMC-Small column (EtOAc/hexanes = 1/2.3 to 1/1, v/v) gave protected glucoxylan hexasaccharide **S2** (5.5 mg, 19% yield over 13 steps) as a glassy solid.

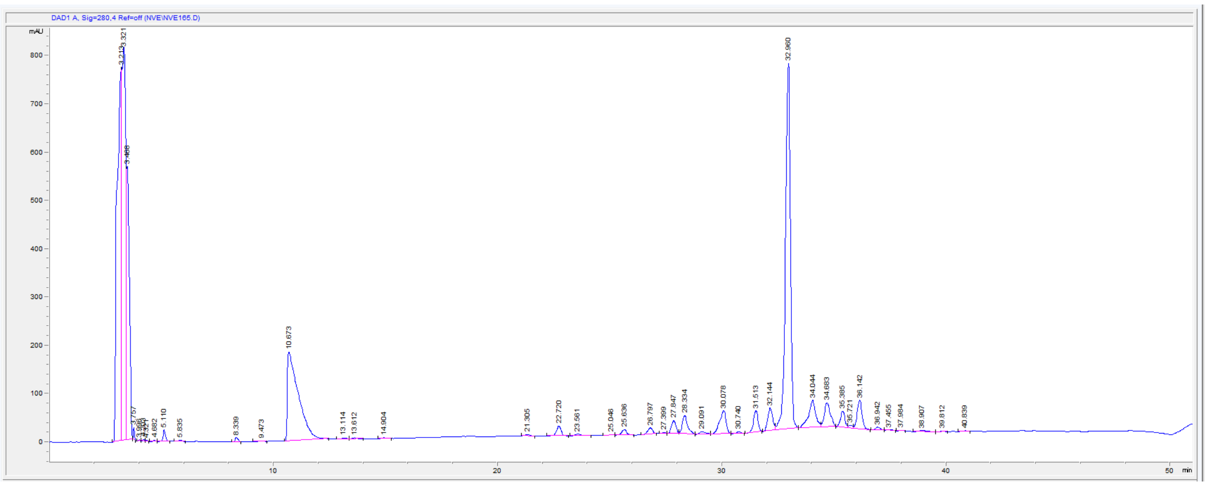

Analytical HPLC of the crude reaction mixture was performed on a YMC-Small NP column using a gradient of EtOAc in hexanes (50 min, flow rate 1 mL/min).

^{1}H NMR (600 MHz, $(CD_3)_2CO$): *δ* 7.89-7.80 (m, 6H, Ar-H), 7.70-7.66 (m, 2H, Ar-H), 7.66-7.63 (m, 2H, Ar-H), 7.61-7.55 (m, 4H, Ar-H), 7.52-7.46 (m, 2H, Ar-H), 7.44-7.18 (m, 52H, Ar-H), 7.17-7.13 (m, 2H, Ar-H), 6.08 (br, 1H, N-H), 5.02 (s, 2H, CH_2-Cbz), 4.97 (dd, *J* = 7.6, 9.1 Hz, 1H), 4.87-4.83 (m, 2H), 4.83-4.78 (m, 3H, H-2A, H-1D, H-2D), 4.78-4.75 (m, 5H), 4.74-4.72 (m, 1H), 4.69-4.63 (m, 4H), 4.63-4.58 (m, 3H), 4.57-4.53 (m, 2H), 4.53-4.49 (m, 3H), 4.48-4.41 (m, 3H, H-1A), 4.02-3.94 (m, 6H, H-3A, H-3D), 3.92-3.87 (m, 2H, H-5A), 3.84 (dd, *J* = 4.2, 11.8 Hz, 1H), 3.74 (t, *J* = 7.3 Hz, 1H), 3.72-3.68 (m, 1H, H-6D), 3.68-3.64 (m, 1H), 3.62-3.52 (m, 3H, OCH_2, H-6D), 3.51-3.43 (m, 3H, H-4D), 3.43-3.39 (m, 1H, H-4A), 3.32-3.27 (m, 2H, H-5A, OCH_2), 3.26-3.09 (m, 6H, H-5B, H-5C, H-5D, H-5E, H-5F), 2.92-2.86 (m, 2H, CH_2-NHCbz), 1.37-1.27 (m, 4H, CH_2), 1.16-1.08 (m, 2H, CH_2) ppm.

^{13}C NMR (151 MHz, $(CD_3)_2CO$): *δ* 165.95, 165.29, 165.23, 165.21, 165.15, 165.04, 157.03, 139.94, 139.78, 139.76, 139.72, 139.69, 139.61, 133.99, 133.88, 133.82, 133.79, 133.75, 133.50, 131.17, 130.95, 130.62, 130.58, 130.56, 130.46, 130.36, 129.35, 129.29, 129.27, 129.19, 129.17, 129.05, 128.96, 128.94, 128.88, 128.79, 128.74, 128.70, 128.62, 128.59, 128.58, 128.54, 128.39, 128.29, 128.28, 128.21, 128.19, 128.12, 128.10, 101.62, 101.59, 100.79, 100.53, 100.13, 99.79, 79.20, 78.66, 78.61, 78.58, 77.14, 76.77, 76.67, 76.34, 76.07, 76.05, 75.53, 75.43, 75.36, 75.34, 75.07, 74.96, 74.87, 74.84, 74.04, 73.76, 73.50, 73.48, 73.05, 72.57, 72.51, 72.41, 71.79, 70.02, 69.35, 66.26, 64.57, 63.72, 62.86, 61.78, 61.50, 41.32, 30.22 (*C*H_2-linker, merged with solvent peak), 29.71 (*C*H_2-linker, merged with solvent peak), 23.71 ppm.

ESI-HRMS: m/z $[M + NH_4]^+$ calcd. for $C_{135}H_{139}N_2O_{34}$: 2331.9204; found 2331.9246.

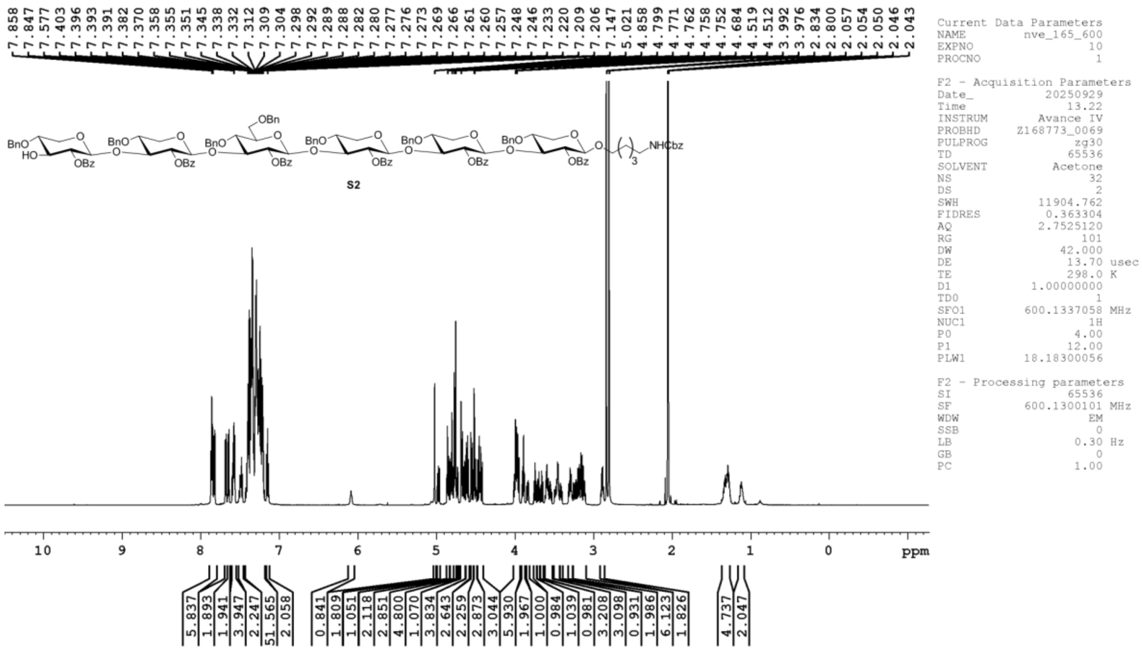

^{1}H NMR spectrum of compound **S2** (600 MHz, $(CD_3)_2CO$)

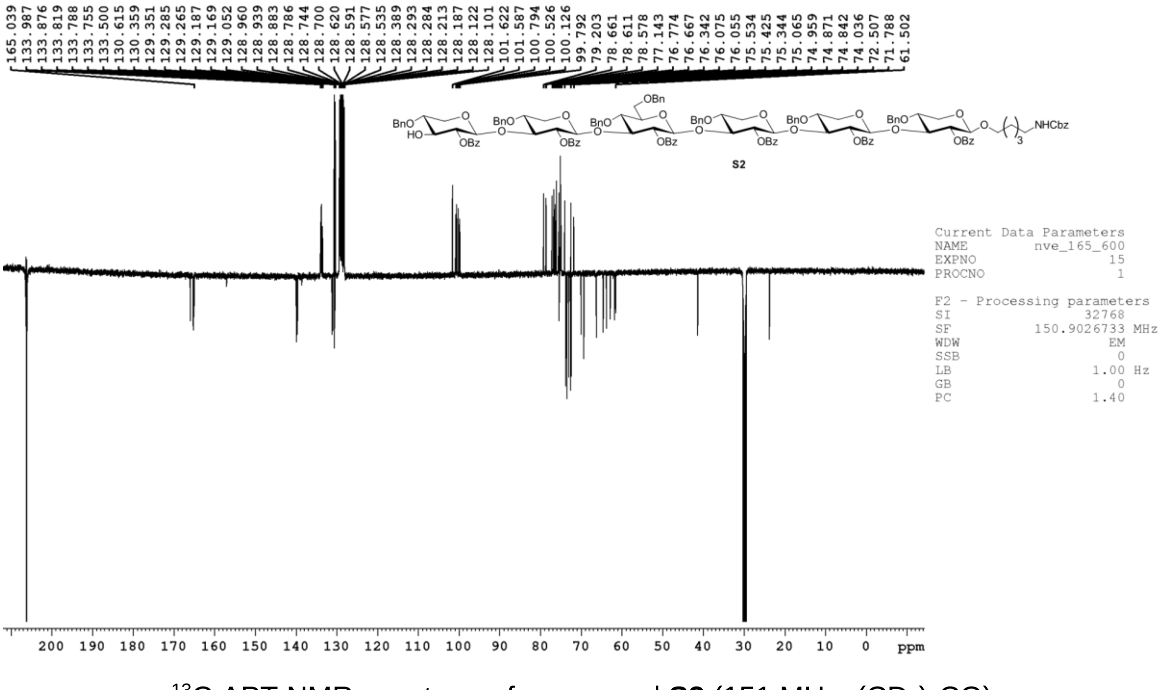

^{13}C APT NMR spectrum of compound **S2** (151 MHz, $(CD_3)_2CO$)

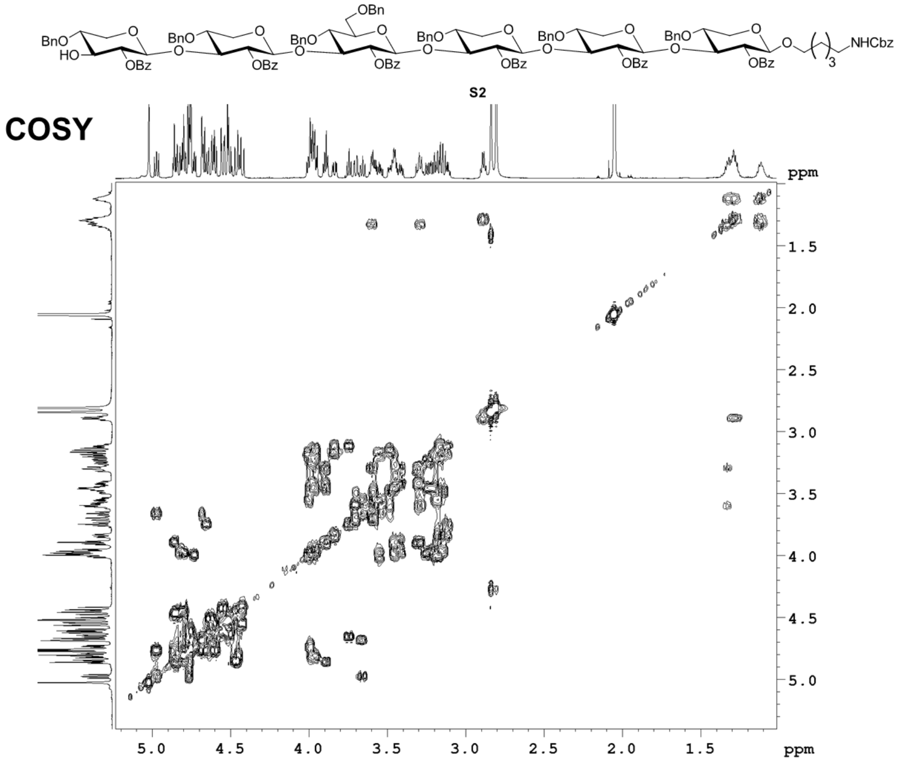

^1H-^1H COSY NMR spectrum of compound **S2** (600 MHz, $(CD_3)_2CO$)

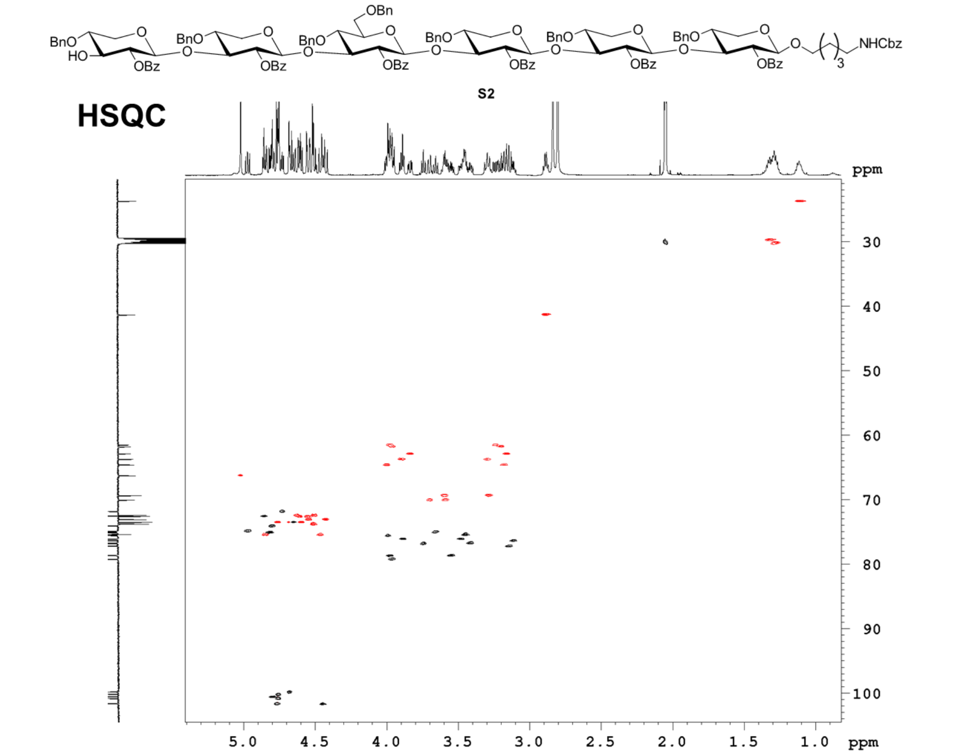

^1H-^{13}C HSQC NMR spectrum of compound **S2** (600/151 MHz, $(CD_3)_2CO$)

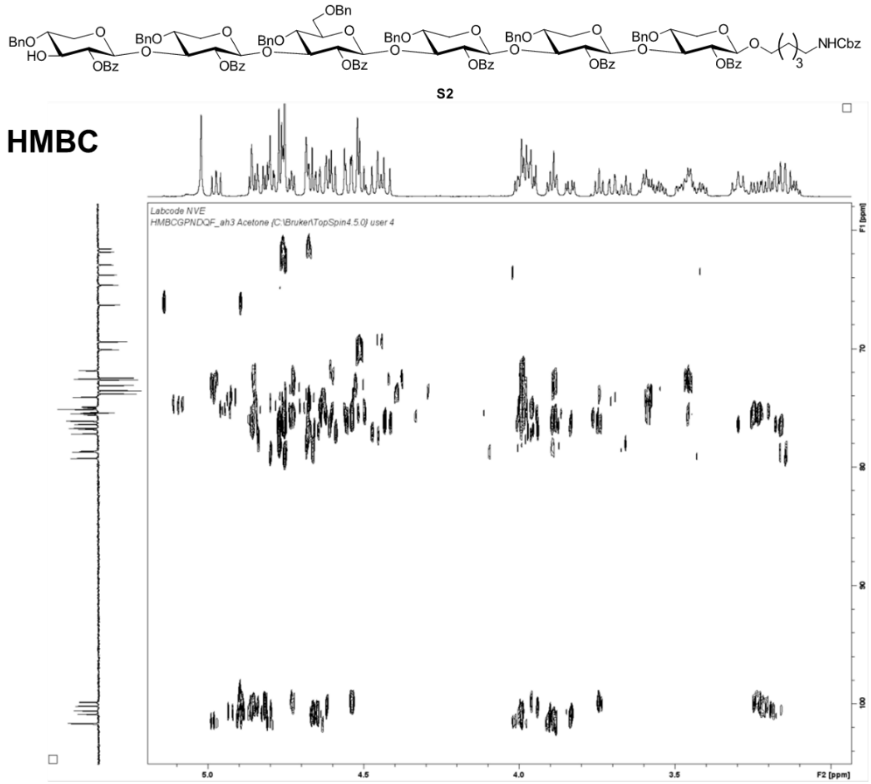

^1H-^{13}C HMBC NMR spectrum of compound **S2** (600/151 MHz, $(CD_3)_2CO$)

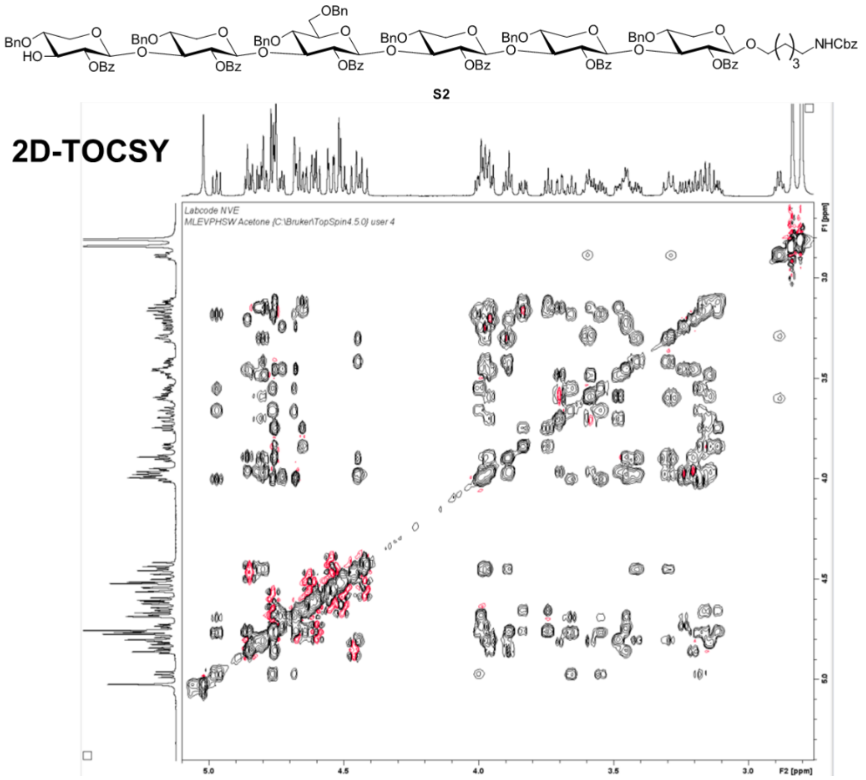

^1H-^1H TOCSY NMR spectrum of compound **S2** (600 MHz, $(CD_3)_2CO$)

Aminopentyl β-D-xylopyranosyl-(1→3)-β-D-xylopyranosyl-(1→3)-β-D-glucopyranosyl-(1→3)-β-D-xylopyranosyl-(1→3)-β-D-xylopyranosyl-(1→3)-β-D-xylopyranoside (2)

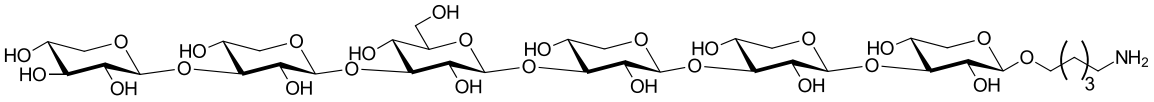

2

Experimental procedure: To a solution of protected hexasaccharide **S2** (5.5 mg, 2.38 µmol) in THF (143 µL, 17 mM), a solution of NaOMe in MeOH (0.5 M, 71 µL, 35.6 µmol) was added at rt, and the reaction mixture was allowed to stir overnight. After confirmation of reaction completion (23 h) by TLC (R_f: 0.6 (MeOH/$CHCl_3$ = 1/15, v/v)), the reaction mixture was neutralized by the addition of IR-120 H^+ resin while slowly stirring the mixture. Then, the reaction mixture was filtered, and the filtrate was concentrated under reduced pressure to yield a partially deprotected glassy crude, which was kept under high vacuum until usage in the next step without any further purification. To a solution of partially deprotected crude (2.38 µmol) in *t*-BuOH (475 µL), H_2O (238 µL), and AcOH (119 µL), unreduced 10% Pd/C (8.5 mg) was added, and the reaction mixture was stirred in the H_2 reactor under a pressure of 8 bar H_2. After 70 h, the reaction mixture was filtered using a PTFE syringe filter (0.45 µm) and concentrated under reduced pressure to yield a crude product, which was purified using pre-packed C18 (500 mg, 6 mL) column chromatography with H_2O/ACN (100% to 80%, v/v, + 0.1% AcOH). The purified product was lyophilized to give glucoxylan hexasaccharide **2** (1.61 mg, 67% yield over 2 steps) as a white amorphous foam.

^{1}H NMR (600 MHz, D_2O): δ 4.76 (1H, merged with D_2O peak, H-1D), 4.72-4.65 (m, 4H, H-1B, H-1C, H-1E, H-1F), 4.41 (d, J = 7.9 Hz, 1H, H-1A), 4.03-3.93 (m, 5H, H-5A, H-5B, H-5C, H-5E, H-5F), 3.92-3.84 (m, 2H, H-6D, OCH_2), 3.75-3.60 (m, 12H, H-6D, OCH_2, H-4A, H-3A, H-4B), 3.55-3.40 (m, 8H, H-2D, H-3B, H-2A), 3.35-3.26 (m, 6H, H-2B, H-5A, H-5B, H-5C, H-5E, H-5F), 2.99 (t, J = 7.6 Hz, 2H, CH_2-NH_2), 1.71-1.61 (m, 4H, CH_2), 1.47-1.40 (m, 2H, CH_2) ppm.

^{13}C NMR (151 MHz, D_2O): δ 104.04, 103.83, 103.77, 103.30, 103.04, 84.38, 84.20, 84.12, 76.31, 76.22, 73.97, 73.94, 73.70, 73.68, 73.63, 73.32, 70.76, 69.80, 68.62, 68.43, 68.28, 68.26, 65.77, 65.44, 65.40, 65.33, 61.31, 39.95, 28.79, 26.99, 22.70 ppm.

ESI-HRMS: m/z $[M + H]^+$ calcd. for $C_{36}H_{64}NO_{26}$: 926.3711; found 926.3728.

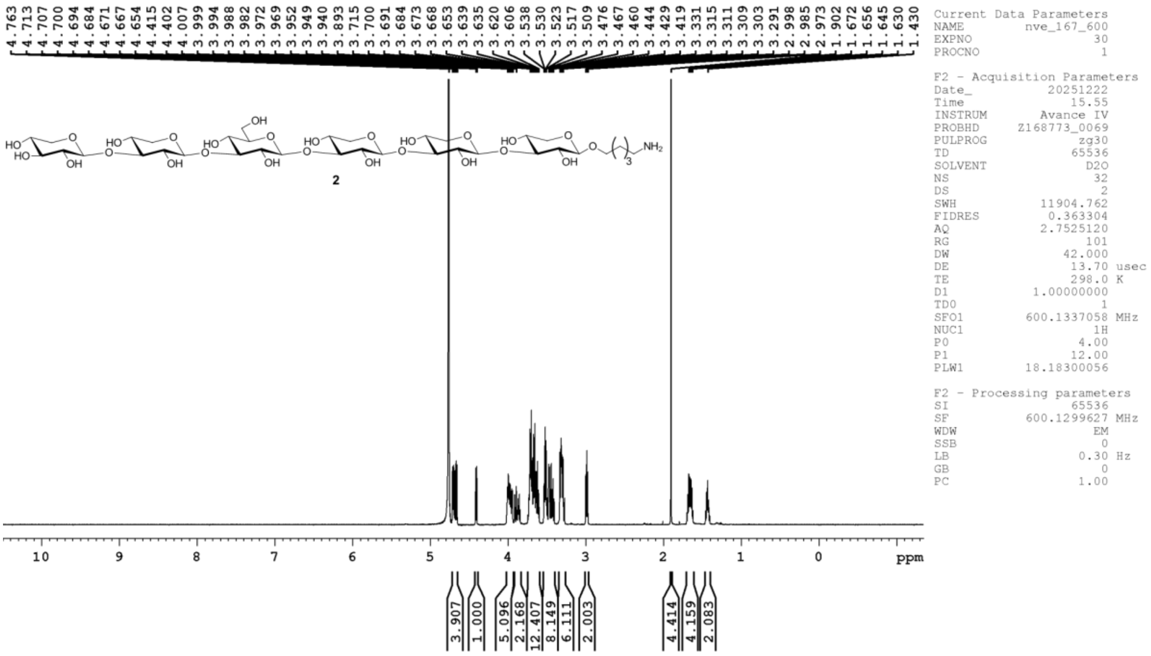

^{1}H NMR spectrum of compound **2** (600 MHz, D_2O)

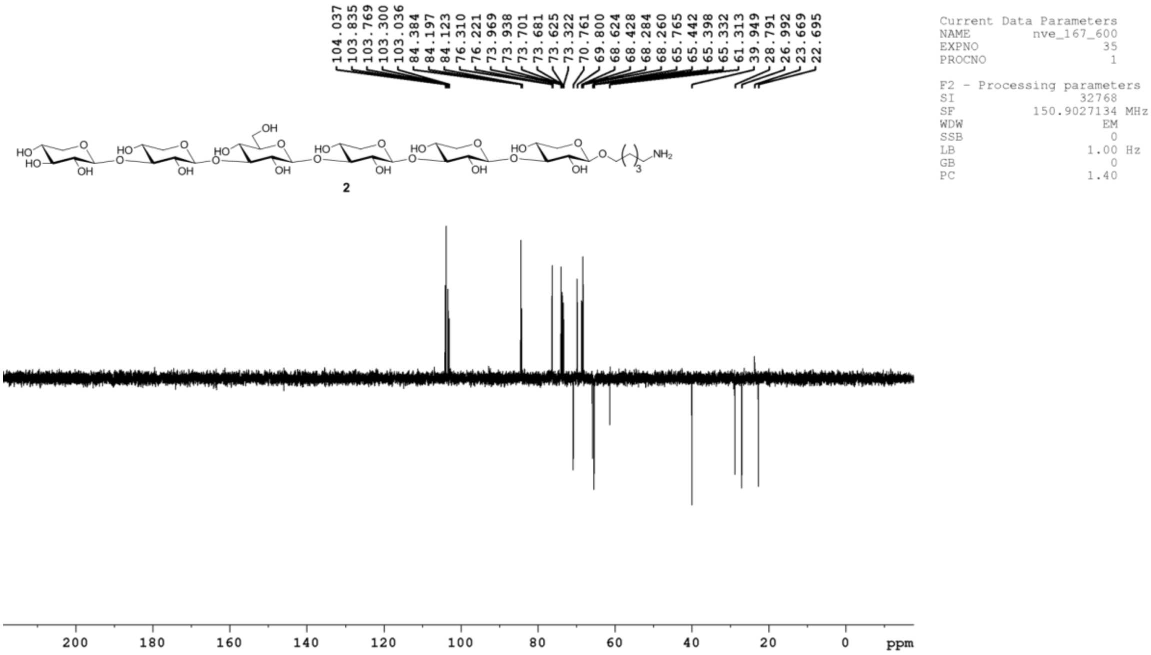

^{13}C APT NMR spectrum of compound **2** (151 MHz, D_2O)

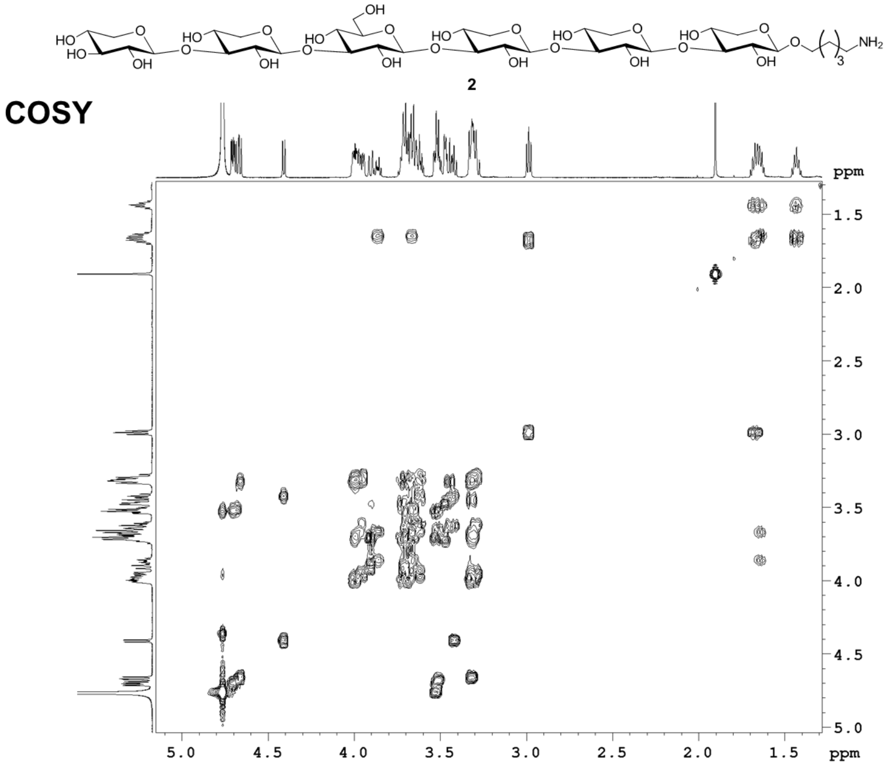

^{1}H-^{1}H COSY NMR spectrum of compound **2** (600 MHz, D_2O)

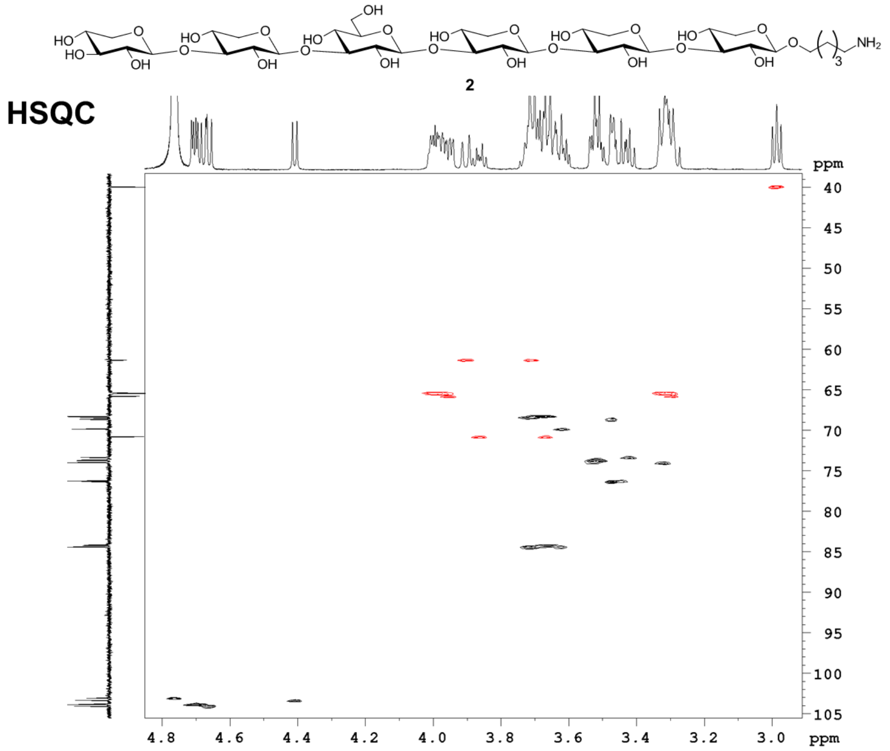

^{1}H-^{13}C HSQC NMR spectrum of compound **2** (600/151 MHz, D_2O)

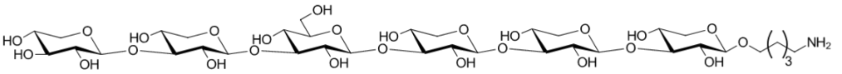

2

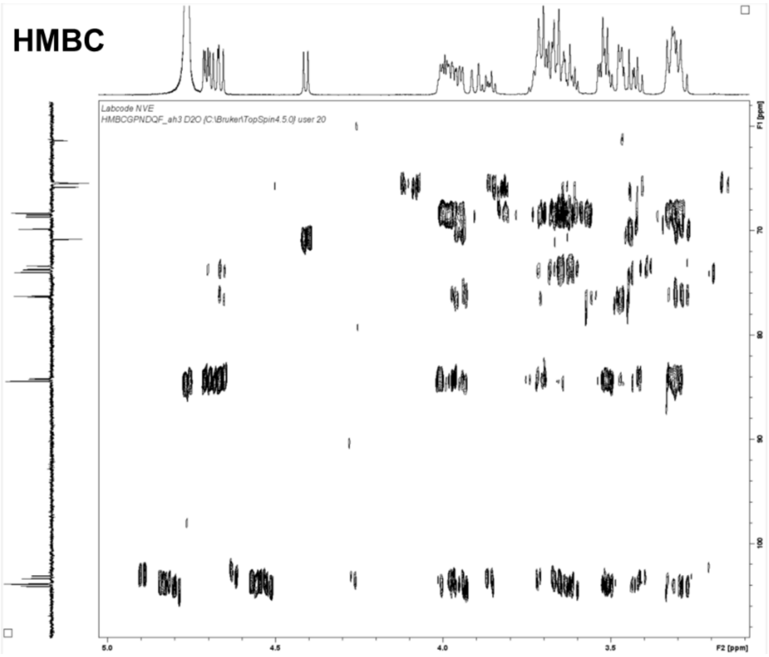

^{1}H-^{13}C HMBC NMR spectrum of compound **2** (600/151 MHz, D_2O)

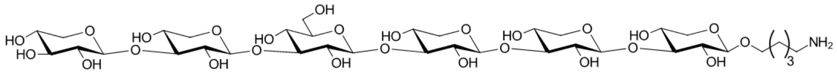

2

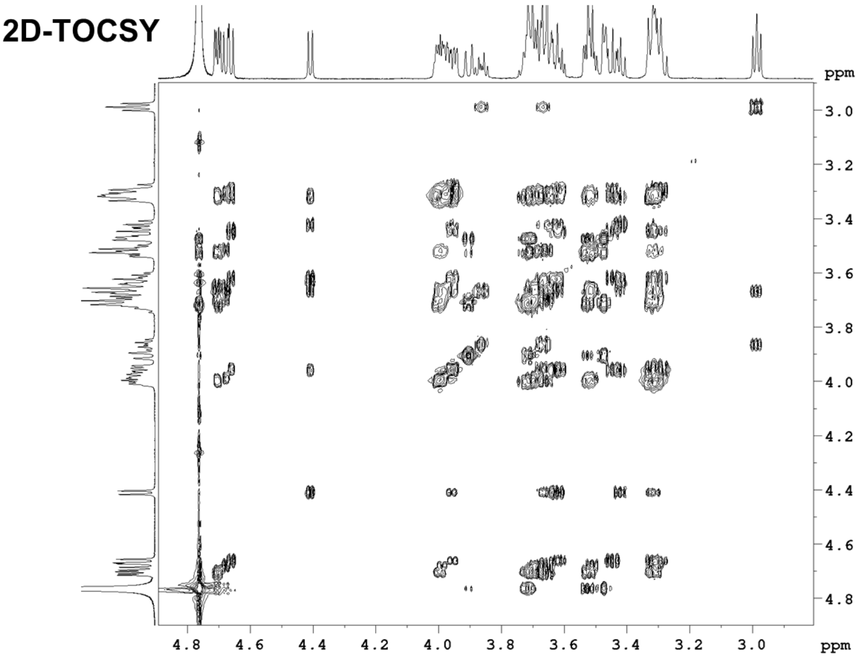

^{1}H-^{1}H TOCSY NMR spectrum of compound **2** (600 MHz, D_2O)

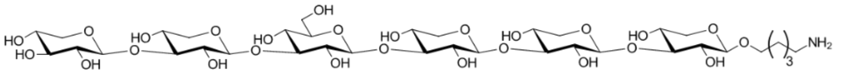

2

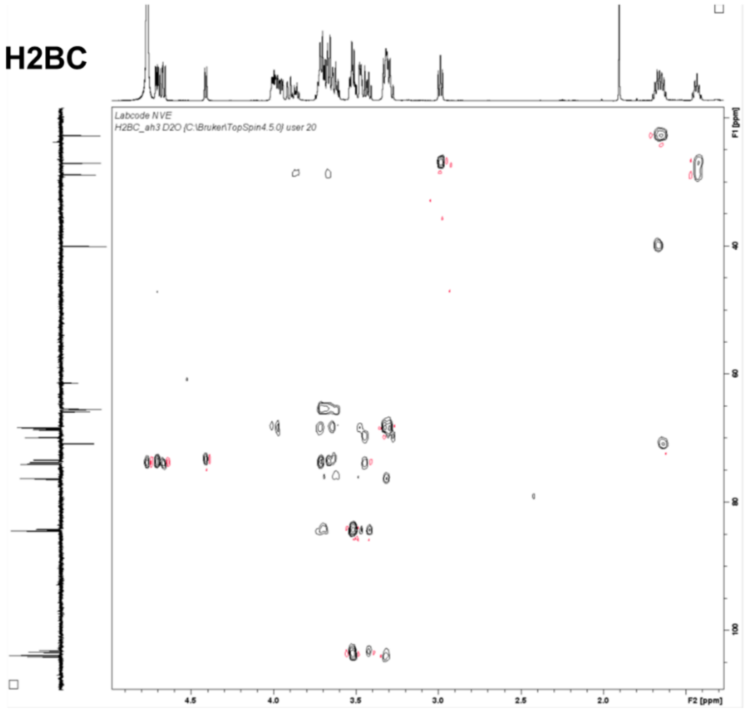

^{1}H-^{13}C H2BC NMR spectrum of compound **2** (600/151 MHz, D_2O)

5.3 AGA of glucoxylan hexasaccharide 3

Benzyloxycarbonylaminopentyl 2-*O*-benzoyl-4-*O*-benzyl-β-D-xylopyranosyl-(1→3)-2-*O*-benzoyl-4,6-di-*O*-benzyl-β-D-glucopyranosyl-(1→3)-2-*O*-benzoyl-4-*O*-benzyl-β-D-xylopyranosyl-(1→3)- 2-*O*-benzoyl-4-*O*-benzyl-β-D-xylopyranosyl-(1→3)-2-*O*-benzoyl-4-*O*-benzyl-β-D-xylopyranosyl-(1→3)-2-*O*-benzoyl-4-*O*-benzyl-β-D-xylopyranoside (S3)

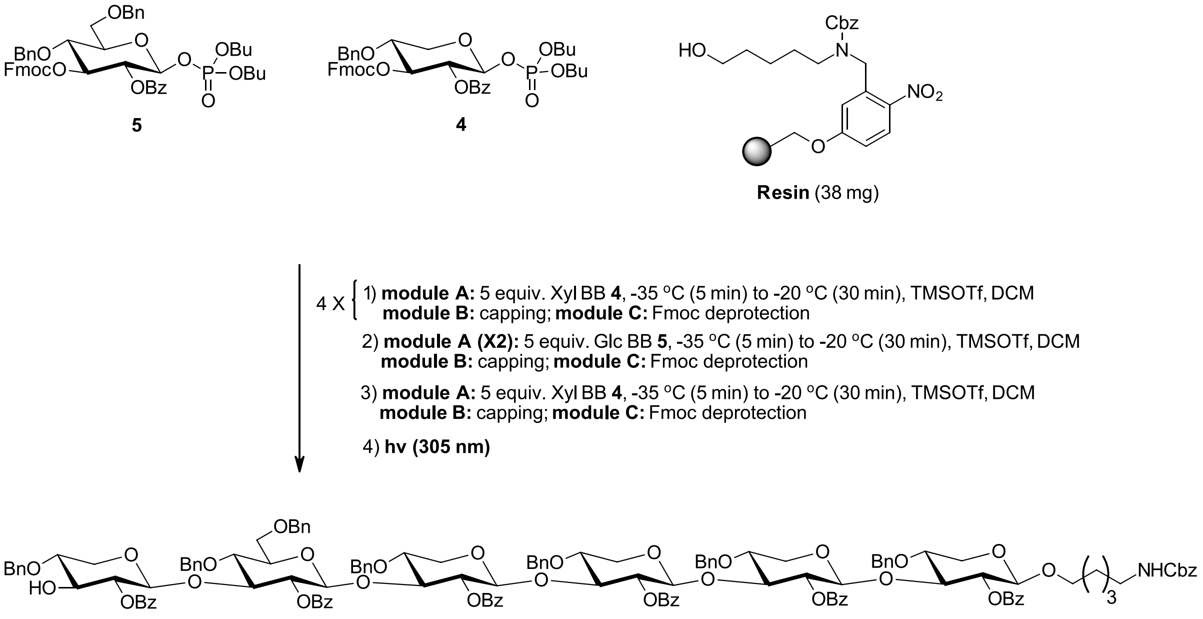

S3

Experimental procedure: Linker-functionalized resin (38 mg, 12.5 µmol) was placed in the synthesizer and synthesizer modules were applied as follows:

4 × [

1) module A (Xylose BB **4**, 47 mg, 0.0625 mmol, 5 equiv.) at –35°C (5 min) to –20°C (30 min), module B, and module C
   ]
2) two cycles of module A (Glucose BB **5**, 55 mg, 0.0625 mmol, 5 equiv.) at –35°C (5 min) to –20°C (30 min), module B, and module C
3) module A (Xylose BB **4**, 47 mg, 0.0625 mmol, 5 equiv.) at –35°C (5 min) to –20°C (30 min), module B, and module C.

Cleavage from the resin using UV irradiation at 305 nm in a continuous flow photoreactor afforded the crude product. Purification of the crude by normal phase HPLC using a preparative YMC-Small column (EtOAc/hexanes = 1/2.3 to 1/1, v/v) gave protected glucoxylan hexasaccharide **S3** (12.3 mg, 43% yield over 13 steps) as a glassy solid.

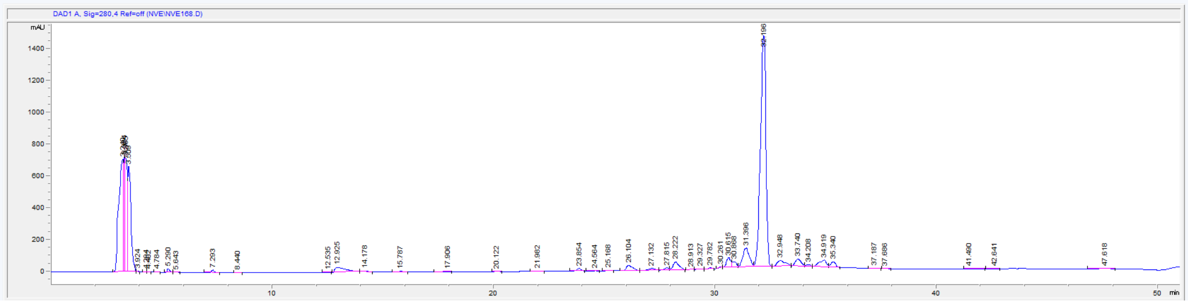

Analytical HPLC of the crude reaction mixture was performed on a YMC-Small NP column using a gradient of EtOAc in hexanes (50 min, flow rate 1 mL/min).

^{1}H NMR (600 MHz, $(CD_3)_2CO$): δ 7.93-7.90 (m, 2H, Ar-H), 7.88-7.85 (m, 2H, Ar-H), 7.78-7.75 (m, 2H, Ar-H), 7.72-7.66 (m, 4H, Ar-H), 7.62-7.56 (m, 5H, Ar-H), 7.51-7.46 (m, 3H, Ar-H), 7.45-7.27 (m, 36H, Ar-H), 7.26-7.14 (m, 16H, Ar-H), 6.08 (br, 1H, N-H), 5.06 (dd, J = 8.0, 9.3 Hz, 1H, H-2E), 5.02 (s, 2H, CH_2-Cbz), 4.98 (d, J = 10.8 Hz, 1H, CH_2-Ph), 4.96 (dd, J = 7.7, 9.0 Hz, 1H, H-2F), 4.86 (d, J = 8.0 Hz, 1H, H-1E), 4.79 (d, J = 5.3 Hz, 1H, H-1B), 4.77-4.62 (m, 12H, H-2A, H-2B, H-2C, H-2D, H-1C, H-1D, H-1F, O-H, CH_2-Ph), 4.61-4.57 (m, 2H, CH_2-Ph), 4.56-4.47 (m, 6H, CH_2-Ph), 4.45-4.41 (m, 2H, H-1A, CH_2-Ph), 4.18-4.11 (m, 1H, H-3E), 4.03-3.94 (m, 5H, H-3D, H-5F, H-5D, H-3A, H-5B), 3.87-3.81 (m, 3H, H-5A, H-3B, H-5C), 3.79-3.74 (m, 2H, H-3C, H-6E), 3.68-3.63 (m, 1H, H-6E), 3.61-3.41 (m, 6H, OCH_2, H-4E, H-5E, H-4F, H-3F, H-4D), 3.36-3.19 (m, 6H, H-4B, H-4A, OCH_2, H-5A, H-5B, H-5D), 3.19-3.07 (m, 3H, H-4C, H-5C, H-5F), 2.93-2.86 (m, 2H, CH_2-NHCbz), 1.36-1.29 (m, 4H, CH_2), 1.16-1.09 (m, 2H, CH_2) ppm.

^{13}C NMR (151 MHz, $(CD_3)_2CO$): δ 165.94, 165.19, 165.16, 165.05, 157.02, 139.96, 139.88, 139.77, 139.75, 139.71, 134.11, 133.89, 133.78, 133.74, 133.58, 133.56, 131.19, 130.93, 130.79, 130.78, 130.68, 130.64, 130.54, 130.48, 130.46, 130.42, 130.33, 129.47, 129.26, 129.24, 129.18, 129.13, 129.04, 129.00, 128.97, 128.94, 128.92, 128.88, 128.74, 128.68, 128.65, 128.62, 128.54, 128.50, 128.31, 128.28, 128.26, 128.22, 128.17, 128.09, 128.02, 101.59, 101.51, 100.81, 100.52, 100.41, 99.93, 79.71, 78.56, 78.52, 77.02, 76.88, 76.70, 76.28, 76.26, 76.15, 76.13, 75.96, 75.49, 75.16, 75.11, 75.08, 75.01, 74.94, 74.91, 74.13, 73.81, 73.54, 73.51, 73.33, 73.08, 72.84, 72.71, 72.55, 72.10, 70.07, 69.29, 66.25, 64.57, 63.76, 62.92, 62.25, 61.80, 41.32, 30.22 (CH_2-linker, merged with solvent peak), 29.71 (CH_2-linker, merged with solvent peak), 23.72 ppm.

ESI-HRMS: m/z $[M + (NH_4)_2]^{2+}$ calcd. for $C_{135}H_{143}N_3O_{34}$: 1174.9771; found 1174.9794.

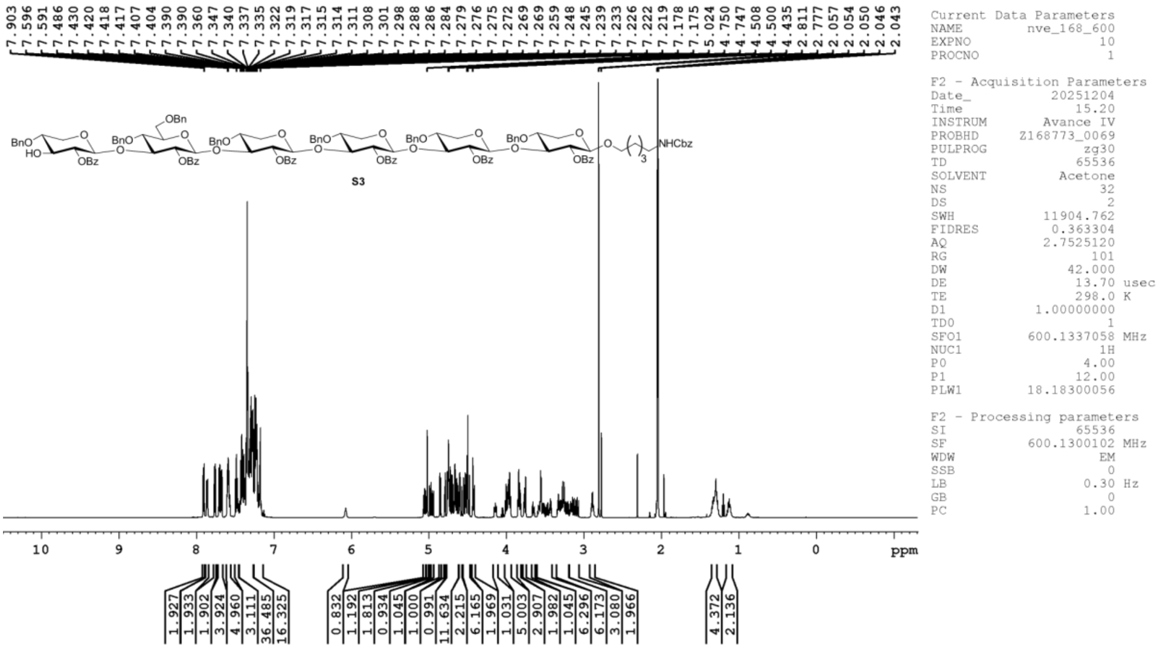

^{1}H NMR spectrum of compound **S3** (600 MHz, $(CD_3)_2CO$)

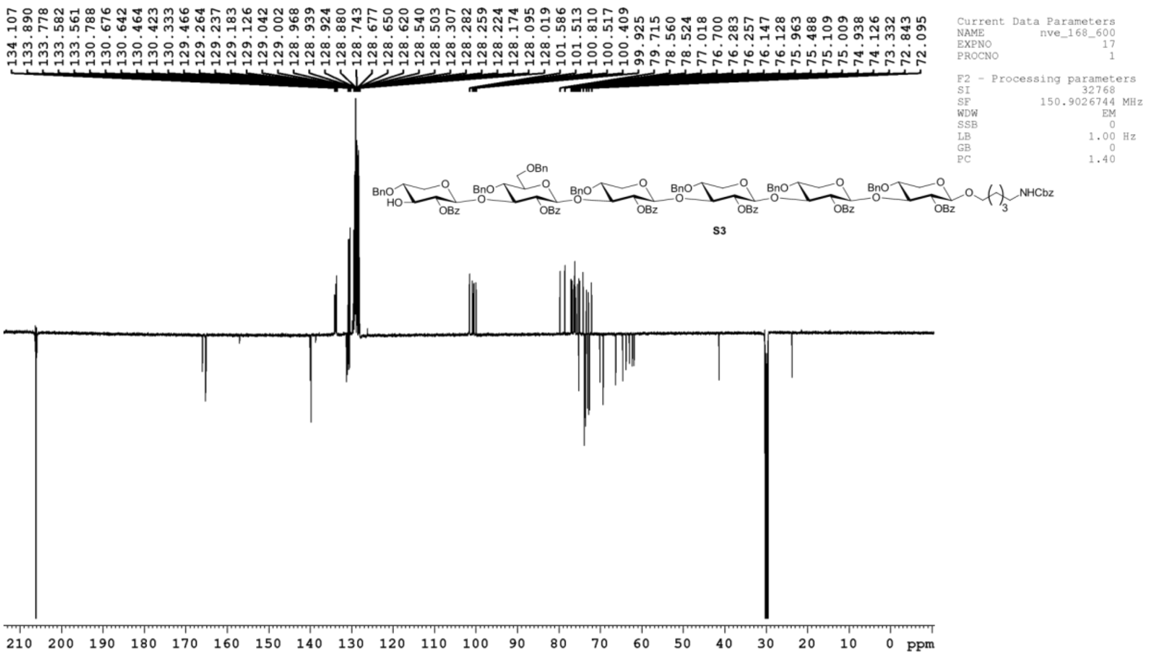

^{13}C APT NMR spectrum of compound **S3** (151 MHz, $(CD_3)_2CO$)

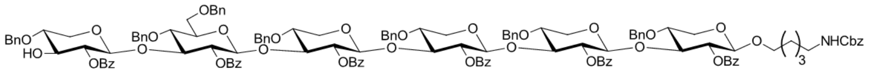

S3

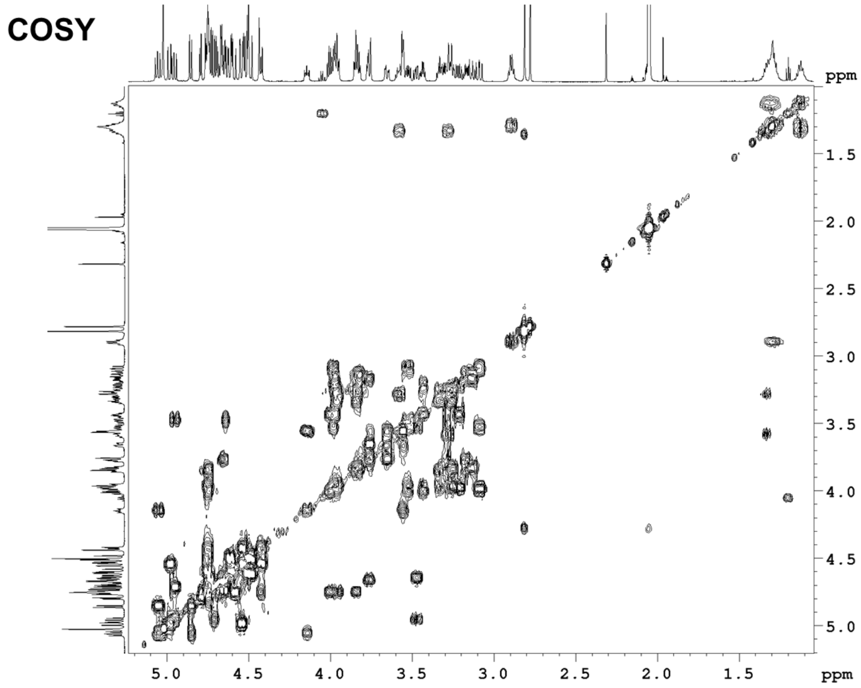

^{1}H-^{1}H COSY NMR spectrum of compound **S3** (600 MHz, $(CD_3)_2CO$)

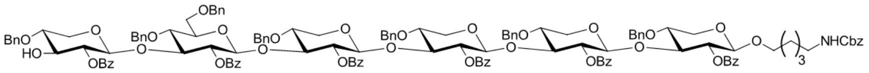

S3

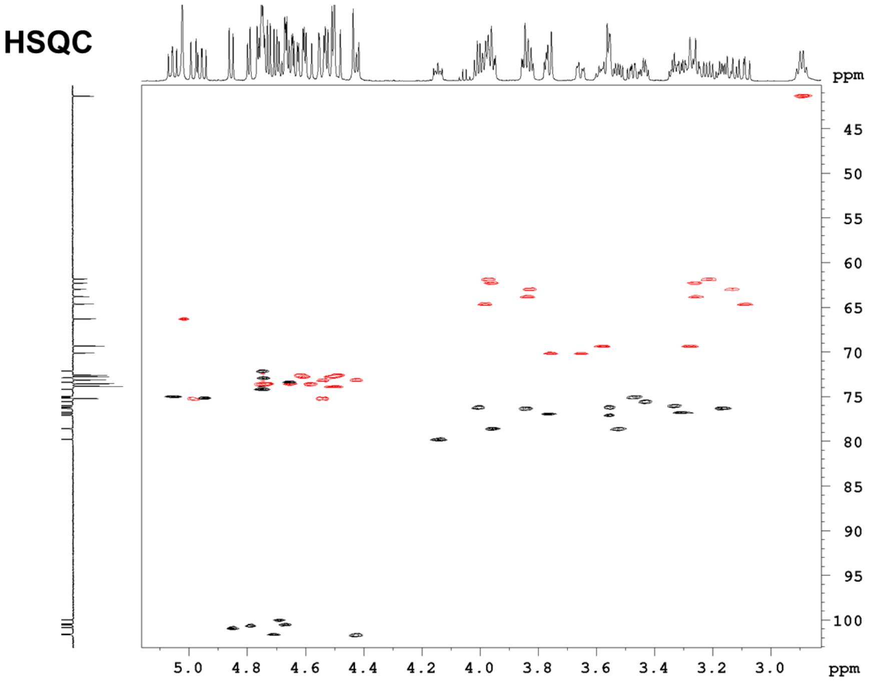

^{1}H-^{13}C HSQC NMR spectrum of compound **S3** (600/151 MHz, $(CD_3)_2CO$)

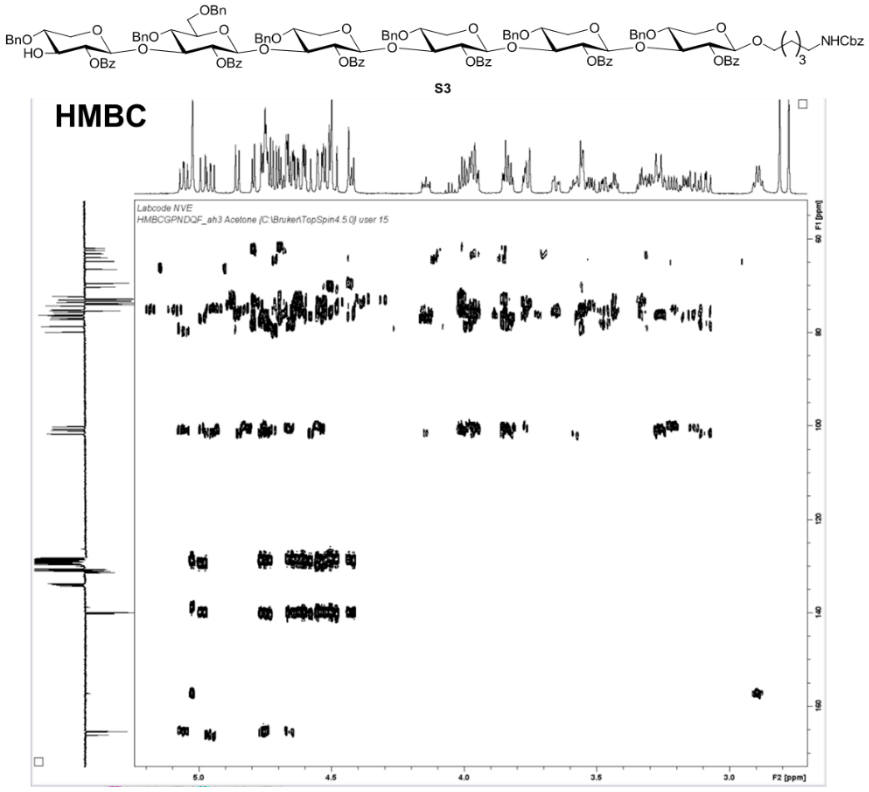

^{1}H-^{13}C HMBC NMR spectrum of compound **S3** (600/151 MHz, $(CD_3)_2CO$)

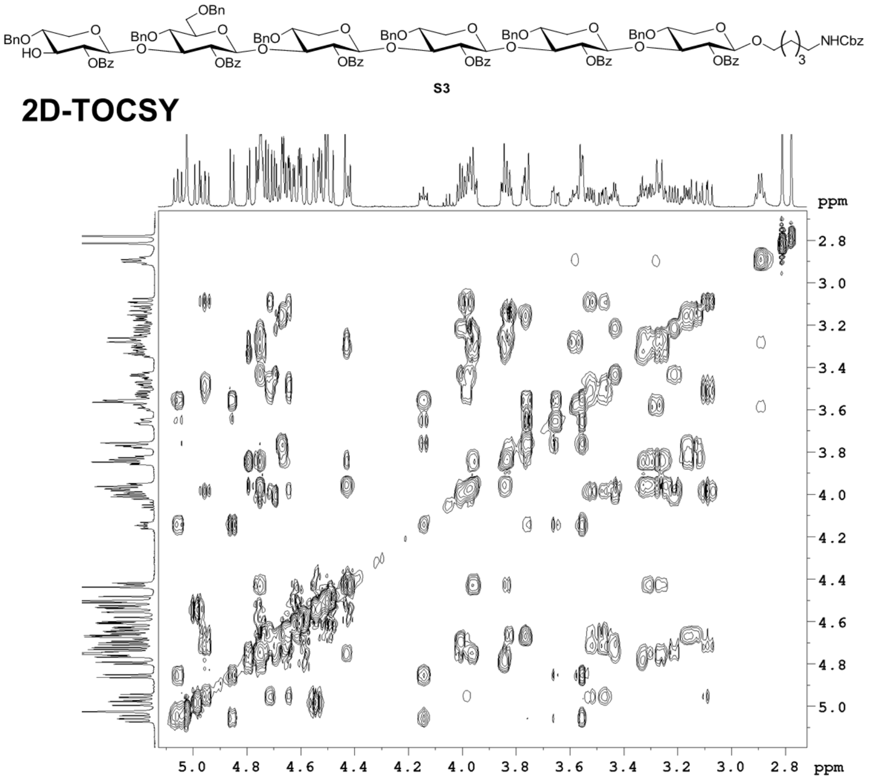

^{1}H-^{1}H TOCSY NMR spectrum of compound **S3** (600 MHz, $(CD_3)_2CO$)

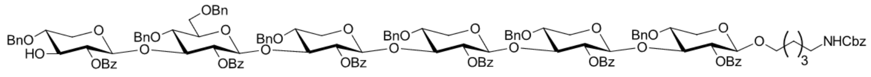

S3

H2BC

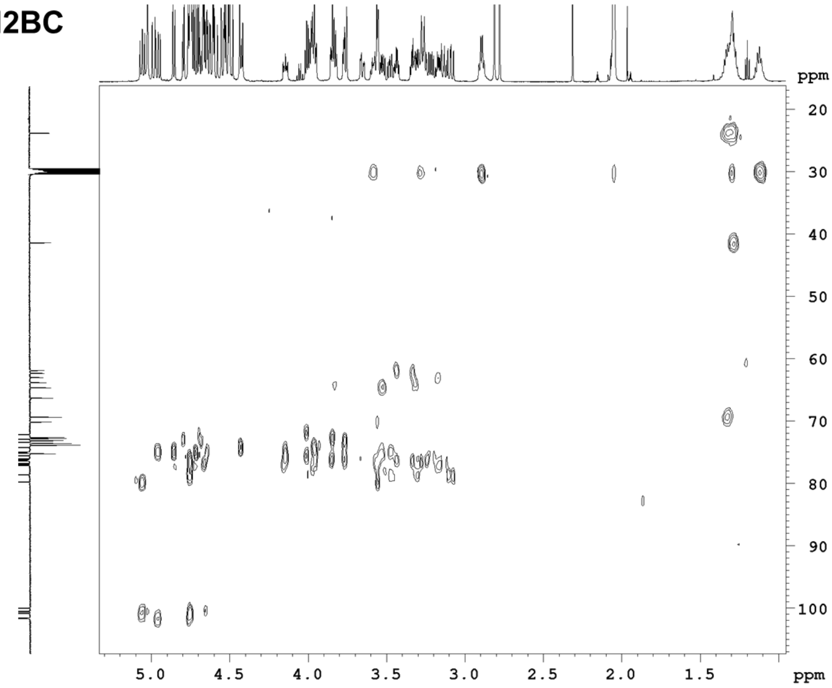

^{1}H-^{13}C H2BC NMR spectrum of compound **S3** (600/151 MHz, $(CD_3)_2CO$)

Aminopentyl β-D-xylopyranosyl-(1→3)-β-D-glucopyranosyl-(1→3)-β-D-xylopyranosyl-(1→3)-β- D-xylopyranosyl-(1→3)-β-D-xylopyranosyl-(1→3)-β-D-xylopyranoside (3)

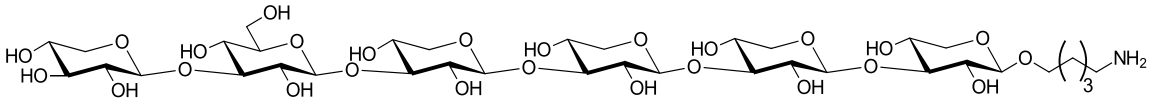

3

Experimental procedure: To a solution of protected hexasaccharide **S3** (12.3 mg, 5.31 µmol) in THF (319 µL, 17 mM), a solution of NaOMe in MeOH (0.5 M, 159 µL, 79.7 µmol) was added at rt, and the reaction mixture was allowed to stir overnight. After confirmation of reaction completion (23 h) by TLC (R_f: 0.54 (MeOH/$CHCl_3$ = 1/15, v/v)), the reaction mixture was neutralized by the addition of IR-120 H^+ resin while slowly stirring the mixture. Then, the reaction mixture was filtered, and the filtrate was concentrated under reduced pressure to yield a partially deprotected glassy crude, which was kept under high vacuum until usage in the next step without any further purification. To a solution of partially deprotected crude (5.31 µmol) in *t*-BuOH (800 µL), H_2O (400 µL), and AcOH (200 µL), unreduced 10% Pd/C (17.3 mg) was added, and the reaction mixture was stirred in the H_2 reactor under a pressure of 8 bar H_2. After 72 h, the reaction mixture was filtered using a PTFE syringe filter (0.45 µm) and concentrated under reduced pressure to yield a crude product, which was purified using pre-packed C18 (500 mg, 6 mL) column chromatography with H_2O/ACN (100% to 80%, v/v, + 0.1% AcOH). The purified product was lyophilized to give glucoxylan hexasaccharide **3** (4.06 mg, 78% yield over 2 steps) as a white amorphous foam.

^{1}H NMR (600 MHz, D_2O): δ 4.77 (1H, merged with D_2O peak, H-1E), 4.72-4.65 (m, 4H, H-1C, H-1D, H-1F, H-1B), 4.41 (d, J = 7.9 Hz, 1H, H-1A), 4.03-3.93 (m, 5H, H-5A, H-5B, H-5C, H-5D, H-5F), 3.93-3.83 (m, 2H, H-6E, OCH_2), 3.74-3.59 (m, 12H, H-6E, OCH_2, H-4A, H-3A, H-4B), 3.55-3.40 (m, 8H, H-2E, H-2A, H-2C, H-2D, H-2F, H-5E, H-3B), 3.35-3.26 (m, 6H, H-2B, H-5A, H-5B, H-5C, H-5D, H-5F), 2.98 (*t*, J = 7.6 Hz, 2H, CH_2-NH_2), 1.71-1.61 (m, 4H, CH_2), 1.46-1.39 (m, 2H, CH_2) ppm.

^{13}C NMR (151 MHz, D_2O): δ 104.11, 103.83, 103.79, 103.77, 103.30, 103.03, 84.53, 84.39, 84.36, 84.15, 84.12, 76.30, 76.21, 73.98, 73.89, 73.70, 73.32, 70.77, 69.80, 68.64, 68.43, 68.26, 65.77, 65.43, 65.40, 65.33, 61.32, 39.96, 28.80, 27.06, 22.70 ppm.

ESI-HRMS: m/z $[M + Na]^+$ calcd. for $C_{36}H_{63}NO_{26}Na$: 948.3531; found 948.3538.

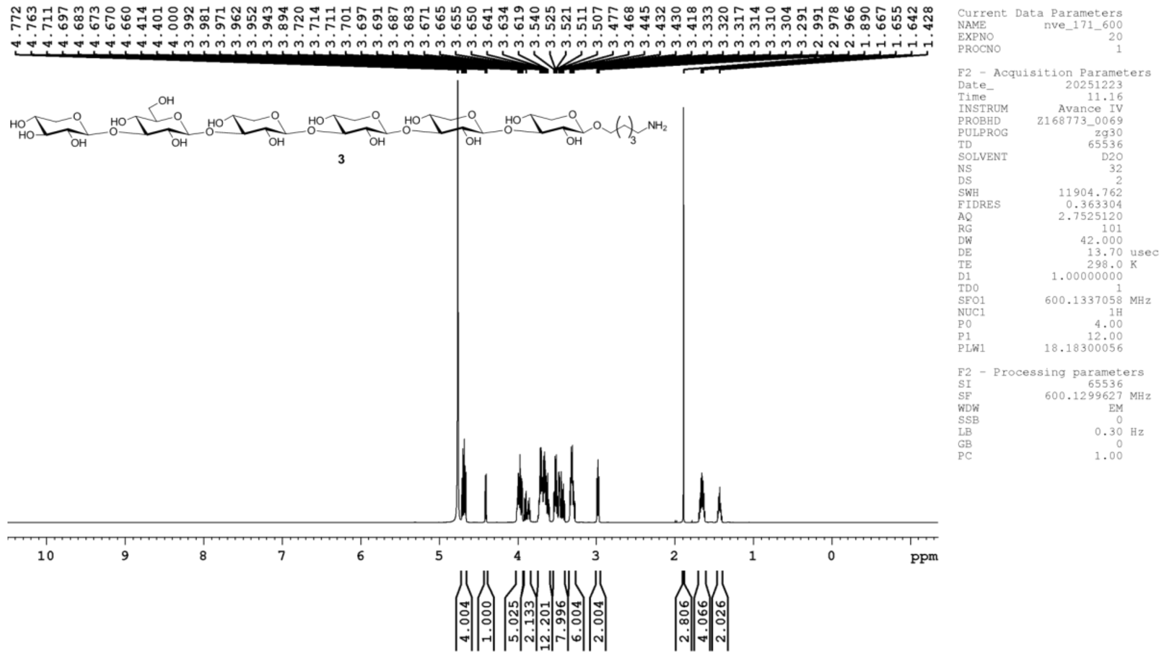

^{1}H NMR spectrum of compound **3** (600 MHz, D_2O)

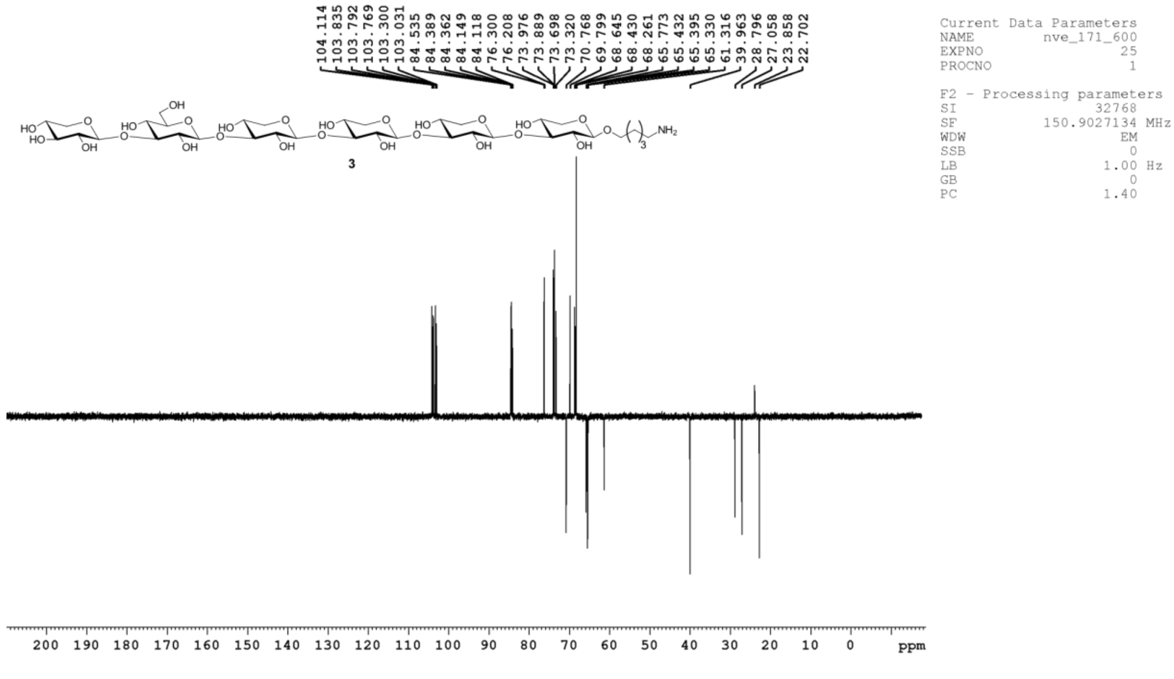

^{13}C APT NMR spectrum of compound **3** (151 MHz, D_2O)

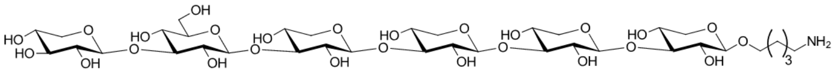

3

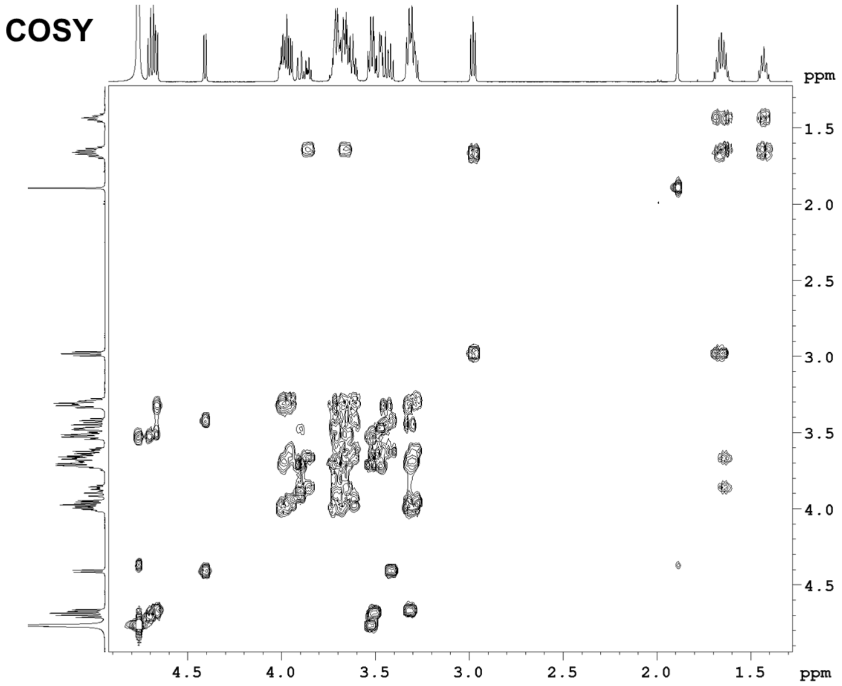

^1H-^1H COSY NMR spectrum of compound **3** (600 MHz, D_2O)

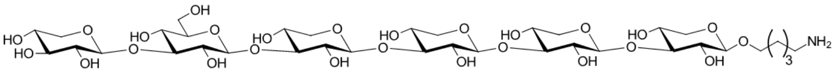

3

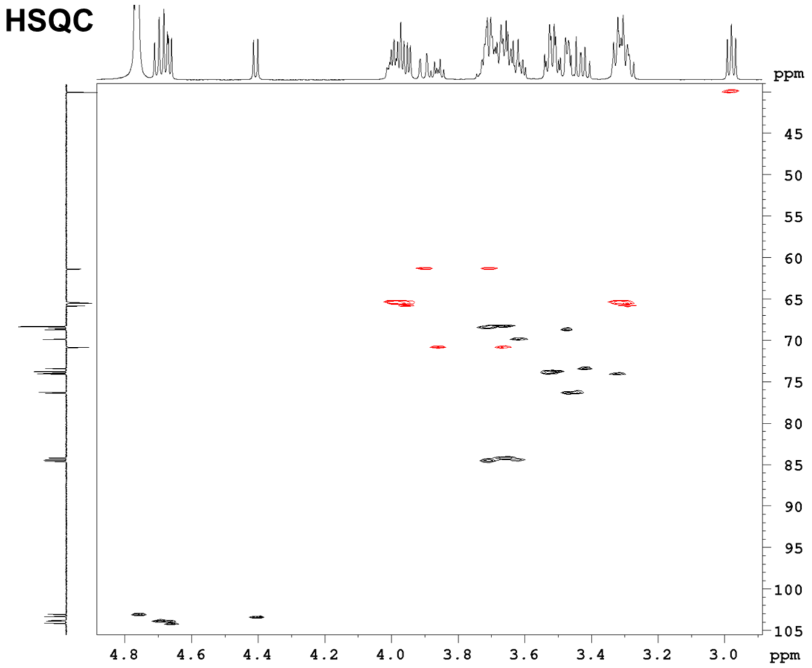

^1H-^{13}C HSQC NMR spectrum of compound **3** (600/151 MHz, D_2O)

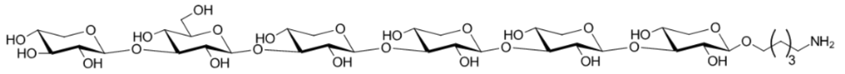

3

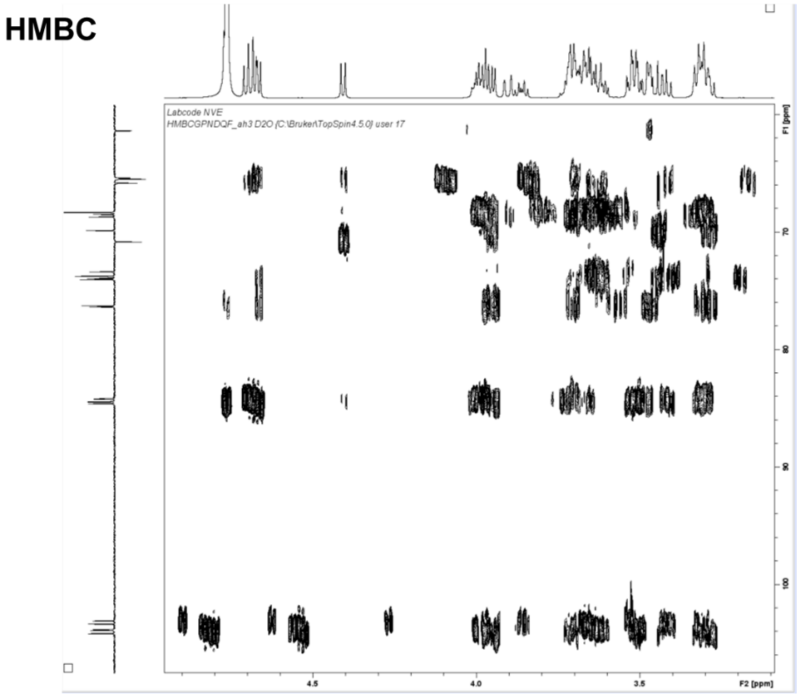

^{1}H-^{13}C HMBC NMR spectrum of compound **3** (600/151 MHz, D_2O)

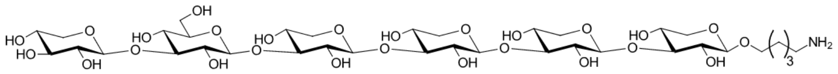

3

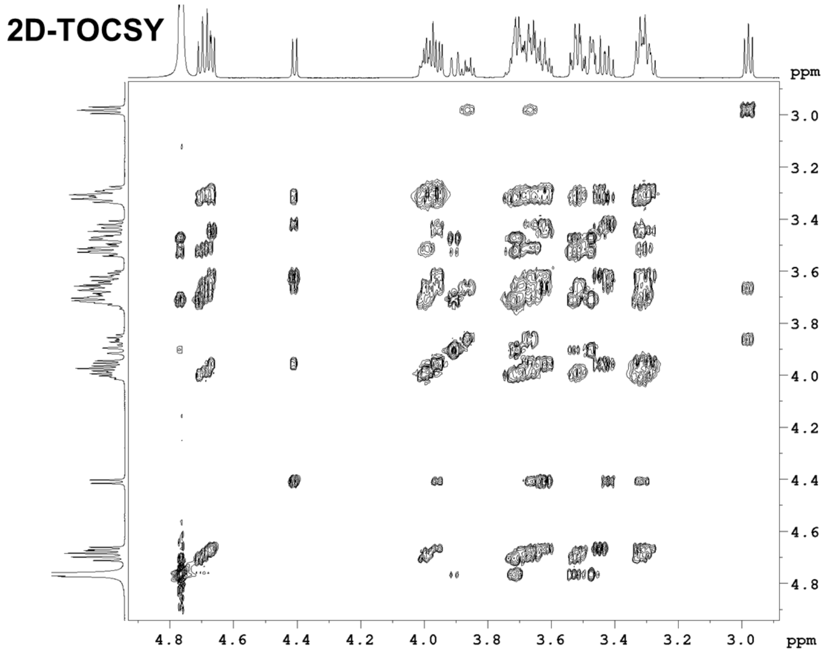

^{1}H-^{1}H TOCSY NMR spectrum of compound **3** (600 MHz, D_2O)

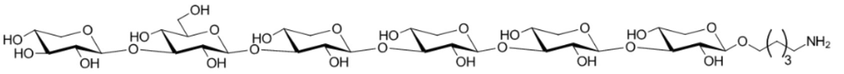

3

H2BC

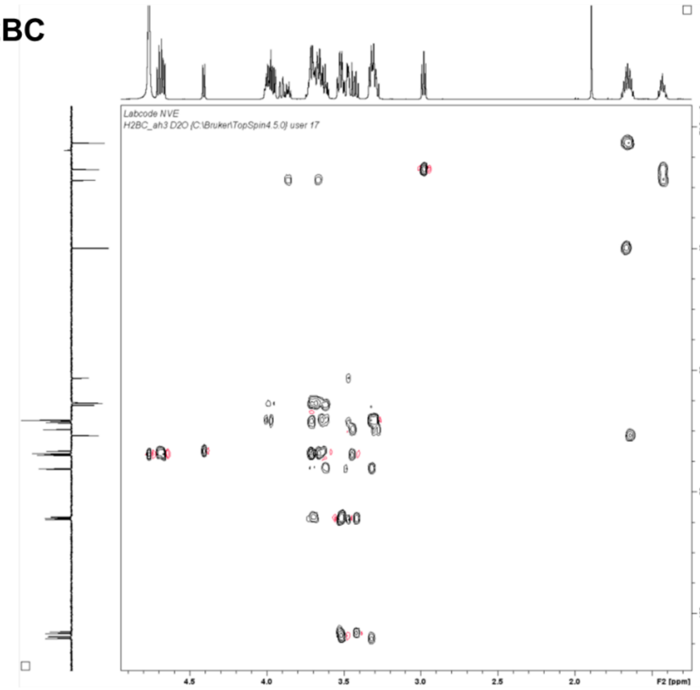

^{1}H-^{13}C H2BC NMR spectrum of compound **3** (600/151 MHz, D_2O)

Table S4. NMR chemical shifts (ppm) of selected proton and carbon atoms in glucoxylan hexasaccharides 1–3.

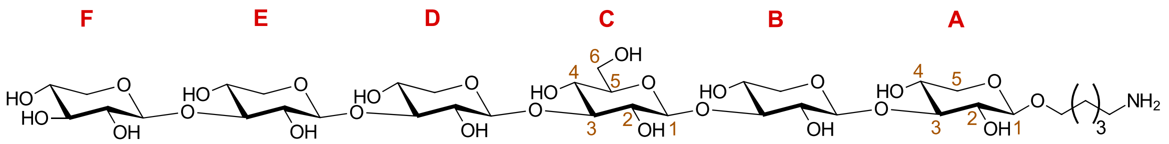

1

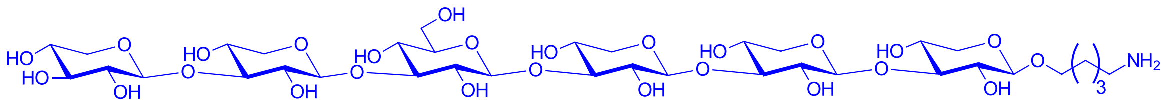

2

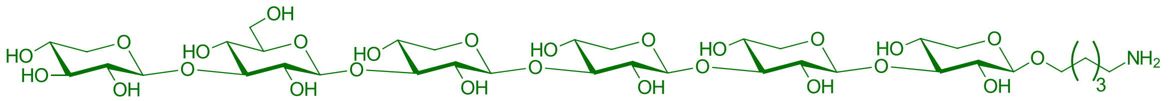

3

	xylose ring A			xylose ring B			glucose ring		
	**1**	**2**	**3**	**1**	**2**	**3**	**1 (C)**	**2 (D)**	**3 (E)**
**H-1 (C-1)**	4.41 (103.304)	4.41 (103.300)	4.41 (103.300)	4.66 (104.042)	4.66 (104.037)	4.66 (104.114)	4.756 (103.040)	4.76 (103.036)	4.77 (103.031)
**H-2 (C-2)**	3.42 (73.328)	3.42 (73.322)	3.42 (73.320)	3.32	3.32 (73.97^{m})	3.31 (73.98)	3.52	3.52 (73.70^{m})	3.52 (73.70^{m})
**H-3 (C-3)**	3.63	3.63 (84.38^{m})	3.63	3.44 (76.223)	3.45 (76.22^{m})	3.44 (76.21)	ND	ND	ND
**H-4**	3.66	3.66	3.66	3.62	3.62	3.63	ND	ND	ND
**H-5a, H-5b (C-5)**	3.96, 3.32	3.96, 3.32	3.96, 3.32	3.96, 3.28 (65.77)	3.96, 3.29	3.96, 3.28	H-5 = 3.47	ND	H-5 = 3.47
**H-6a, H-6b (C-6)**	NA	NA	NA	NA	NA	NA	3.90, 3.71 (61.308)	3.90, 3.71 (61.313)	3.90, 3.71 (61.316)

ND: not defined; NA: not applicable; m: merged with other peaks.

5. References

These references have also been cited in the main file:

[1] N. Verma, N. Rustmeier, U. Osswald, F. Pfrengle, "Automated Syntheses of Xylan Oligosaccharides Containing *β*3-Linkages Enable Substrate Specificity Studies of Xylanases from Marine Bacteria" *Organic Letters* **27**, (2025): 13565-13570.

[2] M. W. Weishaupt, H. S. Hahm, A. Geissner, P. H. Seeberger, "Automated Glycan Assembly of Branched *β*-(1,3)-Glucans to Identify Antibody Epitopes" *Chemical Communications* **53**, (2017): 3591-3594.
